# High-Throughput Analysis Reveals miRNA Upregulating α-2,6-Sialic Acid through Direct miRNA–mRNA Interactions

**DOI:** 10.1021/acscentsci.2c00748

**Published:** 2022-11-09

**Authors:** Faezeh Jame-Chenarboo, Hoi Hei Ng, Dawn Macdonald, Lara K. Mahal

**Affiliations:** Department of Chemistry, University of Alberta, Edmonton, Alberta T6G 2G2, Canada

## Abstract

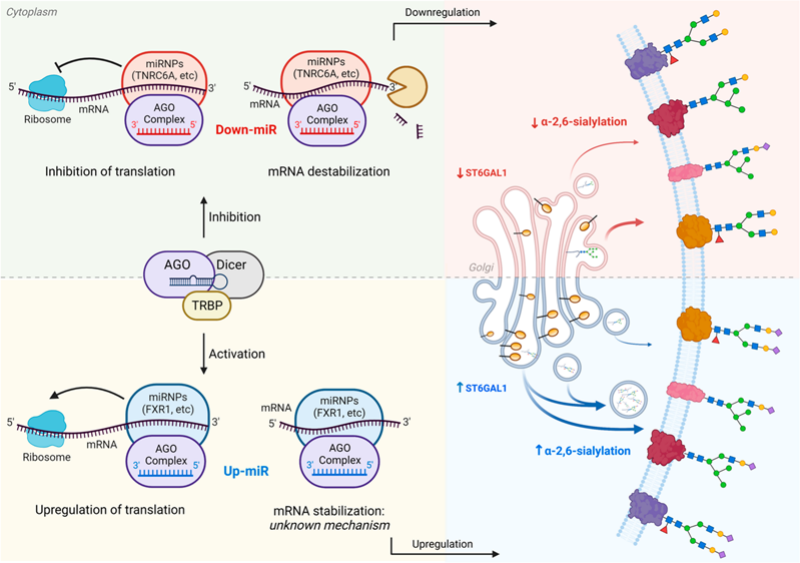

Chemical biology has revealed the importance of sialic acids as a major signal in physiology and disease. The terminal modification α-2,6-sialic acid is controlled by the enzymes ST6GAL1 and ST6GAL2. Dysregulation of this glycan impacts immunological recognition and cancer development. microRNAs (miRNA, miR), noncoding RNAs that downregulate protein expression, are important regulators of glycosylation. Using our recently developed high-throughput fluorescence assay (miRFluR), we comprehensively mapped the miRNA regulatory landscape of α-2,6-sialyltransferases ST6GAL1 and ST6GAL2. We found, contrary to expectations, the majority of miRNAs upregulate ST6GAL1 and α-2,6-sialylation in a variety of cancer cells. In contrast, miRNAs that regulate ST6GAL2 were predominantly downregulatory. Mutational analysis identified direct binding sites in the 3′-untranslated region (UTR) responsible for upregulation, confirming it is a direct effect. The miRNA binding proteins AGO2 and FXR1 were required for upregulation. Our results upend common assumptions surrounding miRNA, arguing that upregulation by these noncoding RNA is common. Indeed, for some proteins, upregulation may be the dominant function of miRNA. Our work also suggests that upregulatory miRNAs enhance overexpression of ST6GAL1 and α-2,6-sialylation, providing another potential pathway to explain the dysregulation observed in cancer and other disease states.

## Abstract 2

Contrary to the dominant model, miRNA upregulates the expression of ST6GAL1/2 enzymes and α-2,6-sialylation. Upregulation requires FXR1 and AGO2 and occurs via a direct miRNA−mRNA interaction.

## Introduction

**Figure 1:**
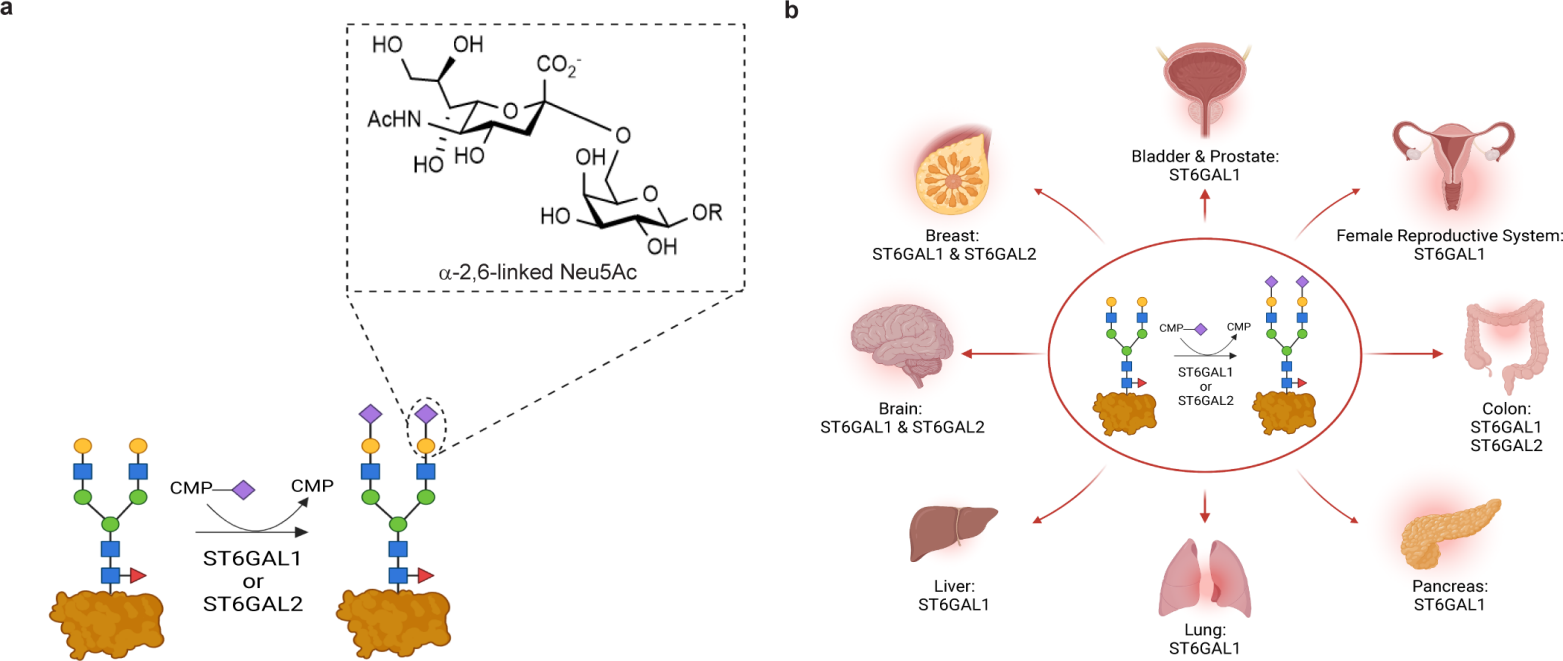
ST6GAL1 and ST6GAL2 are responsible for α-2,6-sialylation. (a) α-2,6-Sialylation by ST6GAL1 or ST6GAL2. (b) Tissue expression map for ST6GAL1 and ST6GAL2.

Chemical biology tools have increasingly revealed the importance of sialic acids as a major signal in physiology and disease.^1−3^ α-2,6-Linked sialic acids on galactose drive cancer development and metastasis,^4−6^ immunological recognition,^7−9^ and microglial phagocytosis.^10,11^ This modification is biosynthesized by two enzymes: ST6-β-galactoside-α-2,6-sialyltranferase-1 (ST6GAL1), expressed throughout the human body, and ST6GAL2, predominantly seen in brain, breast, and colon (Figure 1).^12,13^ Although this modification is critical in both health and disease, the regulation and dysregulation of these enzymes and thus α-2,6-linked sialic acid are poorly understood.

Glycosylation is the outcome of a complex regulatory network in which biosynthetic enzymes can be modulated at the transcriptional, post-transcriptional, translational, and post-translational levels.^14^ Work from our laboratory established miRNA (microRNA, miR) as a major regulator of glycosylation.^15−19^ miRNAs are important modifiers of protein expression, engaging with the 3′-untranslated region (3′-UTR) of mRNA to tune expression levels.^20^ The canonical view of this interaction is that it represses protein levels through the formation of RISC complexes comprised of Argonautes (AGOs), a variety of RNA binding proteins, mRNA and miRNA.^21−23^ The exact nature and number of these complexes are unknown.^22,23^ Repression can occur through either destabilization of the message or inhibition of translation itself. In nondividing cells (e.g., senescent cells, oocytes) and in mitochondria, select cases of miRNA activation of protein expression via direct interactions with the 3′-UTR have also been observed.^24−26^ This upregulation is thought to require destabilized mRNA lacking a 5′-cap and a typical poly(A) tail.^26,27^ These conditions are not met by typical mRNA in actively dividing cells, such as cancer cells, and upregulation of protein expression by miRNA is not thought to occur under these circumstances. Here we show that, in contrast to current assumptions, miRNA can upregulate protein expression, and corresponding glycosylation, in proliferating cancer cells.

We recently established a high-throughput fluorescence assay, miRFluR, that enables interrogation of the entire cohort of human miRNA against the 3′-UTR of a single gene.^28^ Our comprehensive analysis showed intriguing hints that upregulation of protein expression might not be limited to nondividing cells. Herein we apply our assay to analyze the regulation of ST6GAL1 and ST6GAL2, the enzymes underlying α-2,6-sialic acid. Our analysis revealed a surprising result, namely, that most miRNAs impacting ST6GAL1 upregulate the enzyme and α-2,6-sialylation in proliferating cells. Upregulatory miRNAs were also observed for ST6GAL2, although in this case it was not their major mode of action. Validation of our results shows that upregulation by miRNA occurs through direct binding between the miRNA and 3′-UTR. Our data challenges our current understanding of miRNA regulation, implying that upregulation is a normal mode of miRNA function. Our data may help explain why α-2,6-sialylation is commonly upregulated in cancer, as miRNAs that upregulate ST6GAL1 are high in cancers, such as pancreatic cancer, that have high levels of α-2,6-sialylation.^29−31^ Overall, our work showcases the power of chemical biology to bring new understanding to fundamental questions.

## Results and Discussion

### miRFluR Analysis of ST6GAL1 Shows Upregulation As the Major Mode of miRNA Action

**Figure 2:**
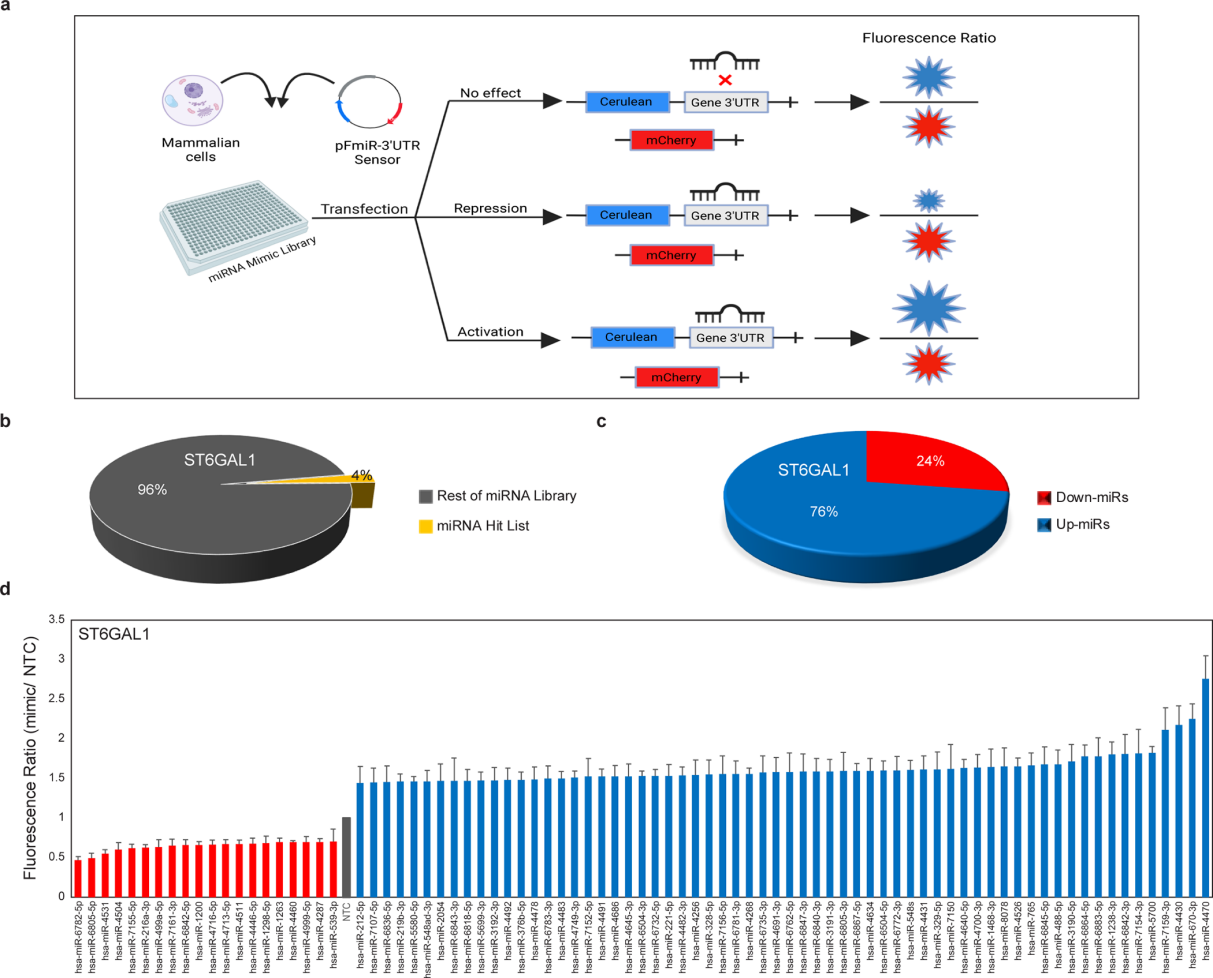
High-throughput analysis for ST6GAL1 reveals miRNA upregulation of expression. (a) miRFluR workflow. (b) Pie chart representing percentage of hits observed from miRNA library for ST6GAL1. (c) Pie chart representing percentage up- vs down-miRs seen in hits. (d) Bar graph of miRNA hits for ST6GAL1. Data are normalized to nontargeting control (NTC). Error bars represent propagated error.

ST6GAL1 is the main enzyme responsible for α-2,6-sialylation throughout the body. To understand the role miRNA might play in ST6GAL1 regulation, we used our miRFluR assay.^28^ This assay uses a genetically encoded ratiometric sensor containing the fluorescent protein Cerulean under the control of the 3′-UTR of the protein of interest and a control fluorophore mCherry (Figure 2a). miRNA downregulating protein expression (down-miRs) would cause a loss of Cerulean signal, and conversely miRNA upregulating protein expression (up-miRs) would cause a gain of Cerulean signal. Our ST6GAL1 sensor contains the most prevalent 3′-UTR for this enzyme (Supporting Information Figure S1). The sensor was transfected into HEK-293T cells along with a human miRNA library in a 384 well format (2601 miRNA mimics, Dharmacon, v21). All miRNA were represented in triplicate. Data was obtained 48 h post-transfection. After quality control (QC, Supporting Information, Materials and Methods), data for 2179 miRNA were obtained. Upon Z-scoring the data, we identified 85 miRNA hits for ST6GAL1 in the 95% confidence interval (Figure 2b–d, Supporting Information Figure S2). This represents 4% of the miRNA tested which passed QC. To our surprise, the majority (76%) of miRNA hits were upregulatory and enhanced protein expression (>1.4-fold) in our assay. Our high-throughput data indicates that upregulation by miRNA may be a common function of these noncoding RNA.

### miRNA Upregulate ST6GAL1 and α-2,6-Sialylation in Cancer Cells

**Figure 3:**
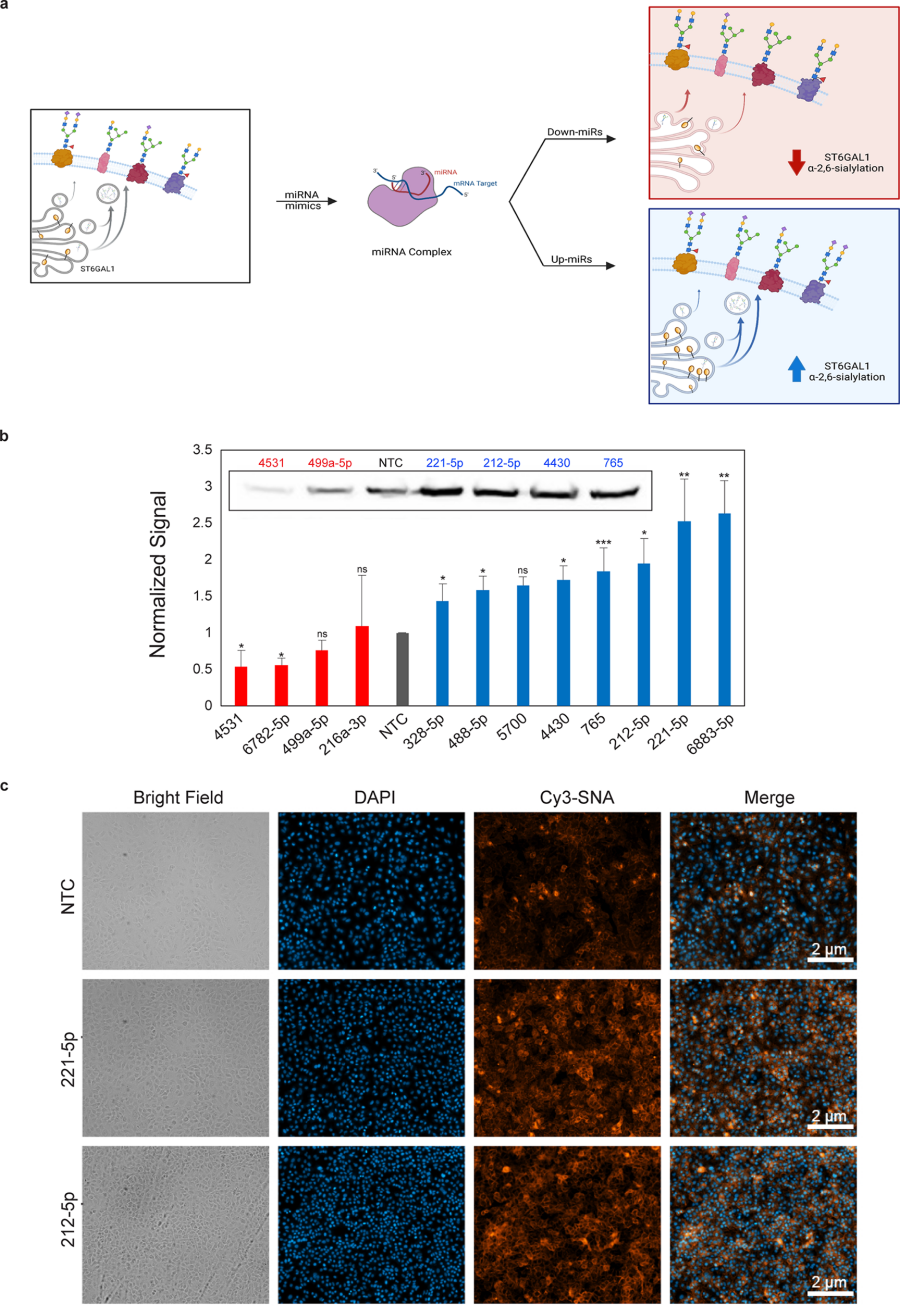
miRNAs regulate α-2,6-sialylation via controlling ST6GAL1 expression at both the mRNA and protein levels. (a) Regulation of ST6GAL1 and α-2,6-sialylation by down- and up-miRs. (b) Quantification of Western blots of ST6GAL1. A549 cells were transfected with miRNA mimics or nontargeting control (NTC, 50 nM, 48 h). Inset shows representative blot. ST6GAL1 expression was normalized by Ponceau and divided by the normalized signal from NTC. miRNAs indicated in figure (blue: up-miR, red: down-miR). (c) SNA staining of up-miR treated cells as in b (NTC, miR-221-5p or miR-212-5p). Additional data including quantification of SNA staining and data in other cell lines are shown in Supporting Information Figures S3–S7. All experiments were performed in biological triplicate. Errors shown are standard deviations. Paired *t* test was used to compare miRs to NTC (^ns^ not significant, * *p* < 0.05, ** < 0.01, *** < 0.001).

To test whether our miRFluR assay is representative of regulation for the actual enzyme ST6GAL1, we validated our findings for a subset of hits (four down-miRs, eight up-miRs). As upregulation was a surprising finding, we validated twice the number of up-miRs and prioritized miRNA with known roles in the literature.^31−34^ To ensure that our findings were reproducible across cell types, we tested four cancer cell lines: A549 (lung, Figure 3 and Supporting Information Figures S3–S4), PANC1 (pancreatic, Supporting Information Figures S5–S6), HT-29 (colon, Supporting Information Figure S7a–d), and OVCAR3 (ovarian, Supporting Information Figure S7e–h). Consistent with previous work, our assay accurately identified regulation of ST6GAL1 by miRNA.^28^ Overall, up- and down-miRs had the anticipated impact on ST6GAL1 protein levels. Although we observed a few cell-specific differences, most notably for downmiRs (miR-499a-5p, miR-216a-3p), in all cases the expected result was observed in at least one cell line, validating the accuracy of our assay. The response at the mRNA level was often discordant with protein levels and showed a high dependency on cell line (Supporting Information Figures S3g, S5d, and S7d,h). This follows data from multiple studies showing discrepancies between mRNA transcript and protein levels.^16,28,35,36^ We also tested the impact of up- and down-miRs targeting ST6GAL1 on α-2,6-sialylation using the α-2,6-sialic acid specific *Sambucus nigra* lectin (SNA).^37^ Our results were consistent with the effects of miRNA on protein expression, with up-miRs increasing and down-miRs decreasing α-2,6-sialic acid levels in both A549 cells (Figure 3c, Supporting Information Figure S4) and PANC1 (Supporting Information Figure S5e–f). Interestingly, the up-miR, miR-221-5p, which has a strong impact on ST6GAL1 and α-2,6-sialylation, is highly expressed in pancreatic cancer and is associated with decreased survival.^34^ In recent work, ST6GAL1 and α-2,6-sialylation have also been shown to be important in pancreatic cancer formation and progression and correlate with decreased survival.^5,6,38^ This points toward a potential functional role for upregulatory miRNA in controlling cancer-related protein and glycan expression.

**Figure 4:**
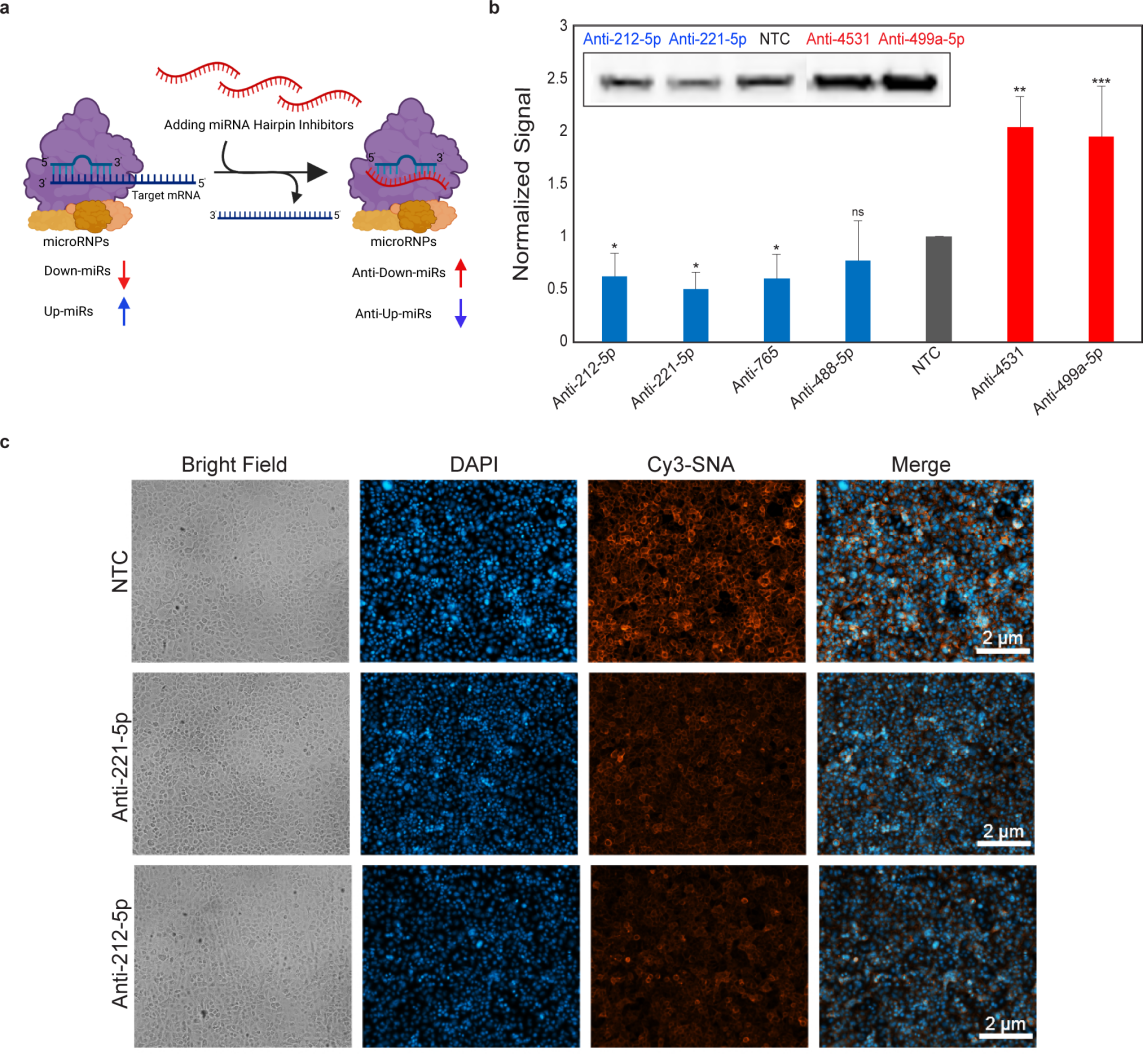
Endogenous miRNA both up- and down-regulate ST6GAL1. (a) Schematic representation of antimiR function. (b) Western blot analysis of ST6GAL1 for indicated antimiRs. A549 cells were transfected with antimiRs or NTC (50 nM, 48 h). Graph represents normalized data for three biological replicates. Inset shows sample Western blot. (c) SNA staining of cells treated as in b with NTC, anti-miR-221-5p and anti-miR-212-5p. Additional data including data in other cell lines are shown in Supporting Information Figures S3–S4, and S6. All experiments were performed in biological triplicate. Errors shown are standard deviations. Paired *t* test was used to compare miRs to NTC (^ns^ not significant, * *p* < 0.05, ** < 0.01, *** < 0.001).

To confirm that the miRNA mimics accurately represent the actions of endogenous miRNA, we utilized antimiRs. These hairpin inhibitors soak up endogenous miRNA, causing relief of repression for down-miRs. Given their mode of action, we would anticipate that anti-miRs of upregulators would cause repression of protein expression (Figure 4a). We tested a subset of antimiRs (4 antiup-miRs: anti-212-5p, -221-5p, -765, -488-5p, and 2 antidown-miRs: anti-4531, -499a-5p) in A549 (Figure 4b–c, Supporting Information Figures S3c,d,h and S4b,d). The antiup-miRs were also tested in PANC1 (Supporting Information Figure S6). All antimiRs chosen had high to moderate levels of miRNA expression in the selected cell lines.^33,39−41^ In accordance with expectation, we found that antiup-miRs downregulated and antidown-miRs upregulated protein expression for ST6GAL1. For antiup-miRs, a concomitant loss of α-2,6-sialic acid was also observed, supporting a function for these miRNAs in maintenance of sialyltransferase and sialylation levels.

### High-Throughput Analysis of ST6GAL2 Shows Predominantly Downregulation by miRNAs

**Figure 5:**
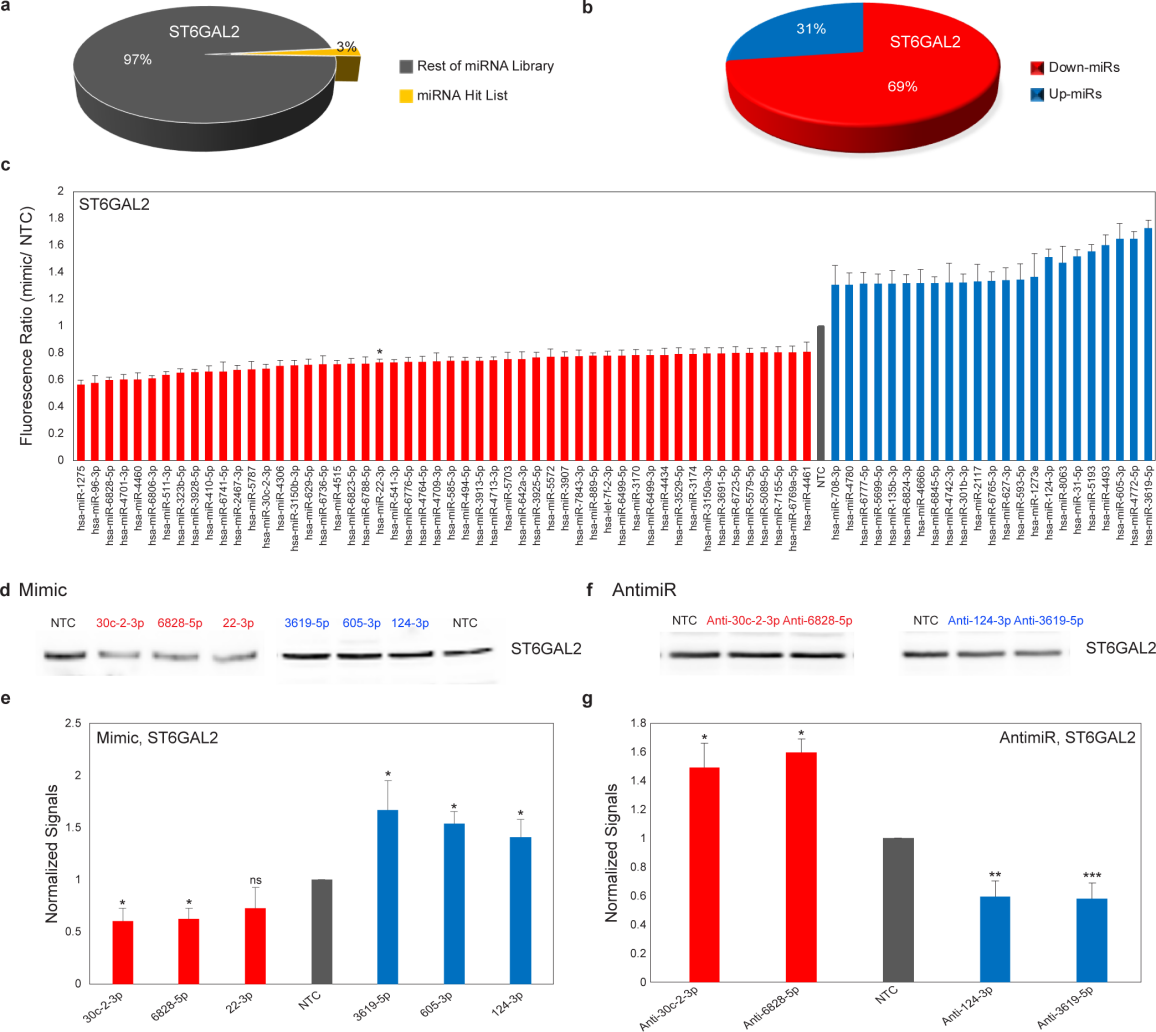
miRNAs regulate ST6GAL2 expression at mRNA and protein levels. (a and b) Pie charts for ST6GAL2 as in Figure 2b–c. (c) Bar graph for ST6GAL2 as in Figure 2d. Star represents a known hit.^42^ (d) Western blot of ST6GAL2. A549 cells were treated with miRNAs as in Figure 3b. (e) Quantitation of Western blot analysis. (f) Western blot analysis of ST6GAL2. A549 cells were treated with antimiRs as in Figure 4b. (g) Quantitative Western blot analysis. Additional data are shown in Supporting Information Figures S10–S11. All experiments were performed in biological triplicate. Errors shown are standard deviations. Paired *t* test was used to compare miRs to NTC (^ns^ not significant, * *p* < 0.05, ** < 0.01, *** < 0.001).

In contrast to ST6GAL1, ST6GAL2 protein expression is restricted to brain, breast, and colon, and its functions as an α-2,6 sialyltransferase are far less studied. We mapped the miRNA regulatory landscape for ST6GAL2, using the most prevalent 3′-UTR for the transcript, in our miRFluR assay (Supporting Information Figure S8). After QC, data for 2166 miRNA were obtained, with 75 miRNA significantly impacting protein expression in our assay (Figure 5a–c, Supporting Information Figure S9). In contrast to ST6GAL1, the majority of hits for ST6GAL2 were downregulatory (69% down-miRs), suggesting the predominant mode of miRNA regulation is target dependent. We validated a subset of miRNA hits in two different cancer cell lines, A549 (Figure 5d–g and Supporting Information Figure S10) and HT-29 (Supporting Information Figure S11). Consistent with our previous work, the impact of miRNA mimics on ST6GAL2 protein expression matched the impact observed using the ST6GAL2-3′-UTR sensor in the miRFluR assay.^28^ We used antimiRs against both up-miRs (anti-3619-5p, -124-3p) and down-miRs (anti-30c-2-3p, -6828-5p) to validate the impact of the endogenous miRNA on ST6GAL2. We again observed that inhibiting endogenous up-miRs caused a loss of protein expression, and inhibiting endogenous down-miRs caused an increase in ST6GAL2 levels. ST6GAL2 has been implicated in breast cancer and is associated with lower survival.^43^ High levels of up-miR-124-3p are also associated with lower survival in breast cancer patients.^44^ This again brings up the possibility that the upregulation observed in cancer may be partially due to miRNA-mediated mechanisms.

### Upregulation by miRNA Is via Direct Interactions with the 3′-UTR and Requires AGO2 and FXR1

**Figure 6:**
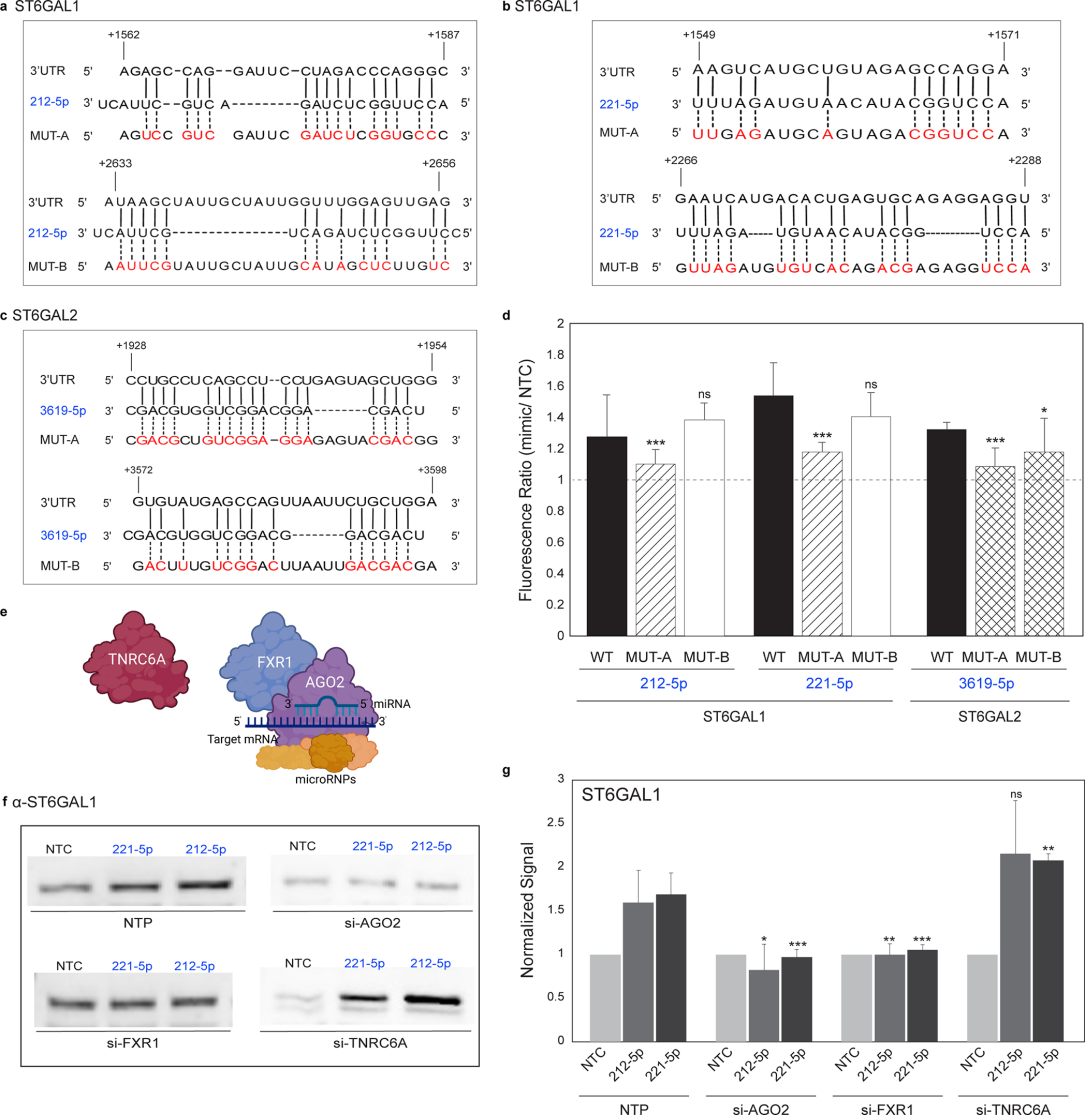
Upregulation of expression by miRNAs requires direct interaction with 3′UTR within a complex containing AGO2 and FXR1. (a and b) Alignment of miRs (a: 212-5p, b: 221-5p) with predicted ST6GAL1-3′-UTR sites and their corresponding mutants. Mutated residues are shown in red. (c) Alignment of miR-3619-5p with predicted ST6GAL2-3′-UTR sites and their corresponding mutants. Mutated residues are shown in red. (d) Bar graph of data from mutant miRFluR sensors as in b and c. Data were normalized over NTC in each sensor. Statistical analysis using the standard *t* test compared the impact of each miRNA in the wild-type (WT) sensor with the corresponding mutant. (e) Schematic representation of potential miRNA complex. (f) Representative Western blot of ST6GAL1. A549 cells were treated with pools of siRNA (nontargeting (NTP), si-AGO2, si-FXR1, si-TNRC6A, 48 h) prior to treatment with miRNA mimics (NTC, miR-212-5p, miR-221-5p, 48 h) and analysis. (g) Quantitative Western blot analysis of experiment shown in f. ST6GAL1 expression normalized as before. All experiments were performed in biological triplicate. Errors are standard deviations. Standard *t* test was used to compare the impact of miRNA in knockdowns with impact in NTP (^ns^ not significant, * *p* < 0.05, ** < 0.01, *** < 0.001). Additional data are shown in Supporting Information Figures S12–S14.

Multiple mechanisms exist by which miRNA could upregulate protein expression. These include regulation by miRNA of gene promoters and enhancers, competition between miRNA, and other indirect effects.^45,46^ Our identification of up-miRs via miRFluR precludes that the observed upregulation of endogenous proteins ST6GAL1 and ST6GAL2 is through miRNA modulation of gene promoter or enhancer elements. To test whether competition between miRNA could explain upregulation, we used RNAhybrid^47^ to identify potential miRNA binding sites for ST6GAL1 and ST6GAL2 (Supporting Information S12a,b). For ST6GAL1, the majority of up-miR sites did not overlap with down-miRs (Supporting Information Figure S12d), arguing that the observed upregulation is not predominantly via miRNA competition. We next tested whether up-miRs act via direct base-pairing. Using RNAhybrid, we identified the most stable potential binding sites and an additional potential site for two up-miRs in ST6GAL1 (miR-212-5p and miR-221-5p, Figure 6a,b) and one in ST6GAL2 (miR-3619-5p, Figure 6c). We mutated all interacting base pairs in the 3′-UTRs to the corresponding miRNA sequence in our miRFluR sensors and tested them against the mimics using our fluorescence assay (Figure 6d). In all cases, the most stable site predicted by RNAhybrid was the binding site for the up-miRs, the mutation of which caused a significant loss of upregulation in comparison with the wild-type sensor (WT). Mutation of down-miR sites (ST6GAL1: miR-4531, ST6GAL2: miR-30c-2-3p) also gave the expected results (Supporting Information Figure S12 e–g). Our data confirm upregulation as a direct effect via binding of the miRNA to the 3′-UTR.

Upregulation of protein expression by direct binding of miRNA to mRNA has been observed in nondividing (quiescent) cells and was thought to require unstable mRNA and AU rich elements.^24−26^ In those cells, Argonaute 2 (AGO2), an important part of the machinery for miRNA-mediated protein repression, and Fragile-X-mental retardation related protein 1 (FXR1) were found to be required for upregulation. However, upregulation was not found in actively dividing cells such as cancer cells. To gain more insight into the requirements for upregulation observed in our work, we classified the predicted sites for our up-miRs on ST6GAL1 and ST6GAL2 by two categories: site motif and AU content. Currently, contiguous binding of at least 6–8 base pairs in the seed region, defined as one base in from the 5′ of the miRNA, is thought to be required for strong binding by RISC complexes which mediate miRNA effects (canonical seed).^21,48,49^ However, noncanonical seed miRNA, in which noncontiguous binding is observed at the 5′ and cases with compensatory interactions at the 3′ of the miRNA can downregulate as well. For up-miRs, the vast majority were predicted to have noncanonical binding and were not AU rich (Supporting Information Figure 12c). Of the three validated up-miR sites, only one had a canonical seed region (miR-221-5p, Figure 6b), and none were AU rich or contained the AU rich element sequence (AUUUA). This contradicts the earlier proposal by Steitz and co-workers^25^ that this motif is required for activation of protein expression by miRNA, although that work was done in quiescent cells.

We next tested whether the upregulation we observe in cancer cells might use the same machinery required for upregulation in quiescent cells. To this end, we used pooled siRNA to deplete FXR1 and AGO2 in A549 cells. FXR1 and AGO2 coimmunoprecipitate and are found in association with one another, although the exact nature of their interactions is currently unknown.^50,51^ We also knocked down the trinucleotide repeat-containing gene 6A (TNRC6A, aka GW182), a scaffolding protein that directly interacts with AGO2 leading to miR-mediated repression.^52,53^ We transfected silenced cells (control (NTP), si-AGO2, si-FXR1, si-TNRC6A) with either nontargeting control (NTC) or individual up-miRs: miR-212-5p or miR-221-5p and examined their impact on ST6GAL1 protein expression. AGO2 and FXR1 were found to be required for upregulation of ST6GAL1 by up-miRs (Figure 6e–g, Supporting Information Figures S13–S14). In contrast, depletion of TNRC6A mildly enhanced upregulation by miR-221-5p compared to the control (∼23% increase, *p* < 0.01), in line with a proposed role for TNRC6A as a repressor.^26^ Similar results were seen with miR-212-5p, but they did not meet the statistical threshold. Overall, this work confirms a role for the FXR1/AGO2 miRNA–protein complexes first identified by Steitz and colleagues in quiescent cells^25^ in upregulation by miRNA in actively dividing cells. This work argues that distinct AGO2 complexes may mediate up- and downregulation by miRNA.

## Conclusion

In many cancers, α-2,6-linked sialic acids are overexpressed, and dysregulation of this glycan is emerging as a crucial part of cancer formation, metastasis, and immune recognition.^4,5,7−9,29^ miRNA are major regulators of the glycome, but their role in controlling α-2,6-linked sialic acid has not been well-studied.^15−19,54^ Our comprehensive analysis of the miRNA regulatory landscape for the α-2,6-linked sialylation enzymes ST6GAL1 and ST6GAL2, described herein, has revealed new potential links between miRNA and the upregulation of α-2,6-linked sialosides observed in cancer.

The dominant view of miRNA regulation is that in proliferating cells the direct impact of miRNA on protein expression is downregulatory. Our high-throughput analysis of ST6GAL1 and ST6GAL2 contradicts this, revealing that upregulatory interactions may be commonplace. Consistent with this, a smaller high-throughput luciferase assay for POT1, PTEN, MXI1, and other cancer-related genes also identified a number of upregulatory miRNA interactions, but these were ignored as noise.^55^ Our previous miRFluR analysis of miRNA-mediated regulation for B3GLCT also identified potential up-miRs for that enzyme.^28^ These interactions have been missed by the scientific community because the current pathway for identifying miRNA interactions has depended on validating potential targets of miRNAs predicted by Targetscan and other algorithms that are focused on downregulation. Recent work knocking out AGO complexes found that removal of the miRNA machinery caused most genes to lose expression, consistent with upregulation being a primary function of miRNA, rather than the expected gain that would come from loss of a repressor.^23^ Taken together, the data support upregulation as part of the broader landscape of miRNA regulatory mechanisms in both dividing and quiescent cells.

For ST6GAL1, which is known to be upregulated in many cancers,^29^ upregulation appears to be the major mode of action of miRNA, although these same miRNAs have downregulatory activity for other genes. Given the importance of miRNA in tuning the expression of genes, it is perhaps unsurprising that regulation by miRNA would be in both directions. Precise control over protein expression is critical for low abundance proteins, where noise becomes an increasing problem.^20^ This class of proteins includes many glycosylation enzymes, GPCRs, and most cell surface receptors. These proteins, which often act as initiators of amplified signals, would be important to tightly regulate. The expanded understanding of the miRNA regulatory landscape provided by our work opens new possibilities for miRNA mechanisms to modulate protein expression and exposes our need to create tools to further explore the impact of these noncoding RNA.

## Notes

All data are available in the main text or the Supporting Information.

## Supporting Information Available

Detailed experimental procedures and additional supporting data including plasmid maps, scatterplots of data, mimics and inhibitors in additional cell lines (Western blots and RT-PCR), additional fluorescence microscopy staining, mapping and analysis of predicted miRNA sites for ST6GAL1 and ST6GAL2, additional experiments with knockdowns (AGO2, FXR1, TNRC6A), table of primers and additional references (PDF)
Data Analysis of miRFluR for ST6GAL1 and ST6GAL2 (XLSX)
