# Supplementary material for: High-Throughput Analysis Reveals miRNA Upregulating α-2,6-Sialic Acid through Direct miRNA–mRNA Interactions
Source: ACS Cent Sci. 2022 Nov 9;8(11):1527–36. doi: 10.1021/acscentsci.2c00748 (PMC9686205; doi:10.1021/acscentsci.2c00748)
Supplement: Supplementary file 1 — oc2c00748_si_001.pdf [file oc2c00748_si_001.pdf]

Supporting Information for

Title: High-throughput analysis reveals miRNA upregulating α-2,6 sialic acid through direct miRNA: mRNA interactions

Faezeh Jame-Chenarboo[1], Hoi Hei Ng[1], Dawn Macdonald[1], Lara K. Mahal[1*]

[1] Department of Chemistry, University of Alberta; Edmonton, Alberta, T6G 2G2, CANADA.

Correspondence to: lkmahal@ualberta.ca

This file includes:

Materials and Methods
Supporting Information Figures S1 to S14
Supporting Information Table S1
Supporting Information Data S1 (additional .xls file)
Supporting References

Total: 26 pages

METHODS

Cloning:

ST6GAL1 and ST6GAL2 3'-UTRs were cloned from genomic DNA (gDNA) extracted from HEK293T cell line using QIAquick gel extraction kit (catalog #: 28706) and primers shown in Table S1. The amplicons were cleaned up using the PCR clean-up kit (catalog #: KTS1115). The 3'-UTR fragments were cloned using the NheI and BamHI restriction sites downstream of Cerulean in the pFmiR-empty backbone [6] using standard ligation protocols (NEB) and verified by Sanger sequencing (Molecular Biology Services Unit, University of Alberta). Large-scale endotoxin free DNA preparations were made for sequence-verified constructs (pFmiR-ST6GAL1 and pFmiR-ST6GAL2) using QIAGEN maxi-prep (catalog #: 12362 and catalog #: 19048). Plasmid maps for pFmiR-ST6GAL1 and pFmiR-ST6GAL2 and the glycogenes' 3'-UTR sequences can be found in Supplementary Fig. 1 and Supplementary Fig. 2, respectively.

Cell Lines:

All cells lines (HEK-293T, A549, PANC1, HT-29, OVCAR-3) were purchased directly from the American Type Culture Collection (ATCC) and cultured using suggested media (HT-29 & HEK-293T: Dulbecco's Modified Eagle Medium (DMEM), 10% FBS; A549: FK-12, 10%, FBS; PANC1: DMEM* (catalog # 30-2002), 10% FBS; OVCAR3: RPMI-1640, 20% FBS with 0.01 mg/mL bovine insulin) under standard conditions (5% CO_2, 37 °C). All cells used were below passage number 15.

miRFluR High-throughput Assay:

The Human miRNA mimic library version 21 (MISSION, Sigma) was resuspended in ultrapure nuclease-free water (REF. #: 10977-015, Invitrogen) and aliquoted into black 384-well, clear optical bottom tissue-culture treated plates (Nunc). Each plate contained three replicate wells of each miRNA in that plate (1.8 pmol/well). In addition, each plate contained a minimum of 6 wells containing non-targeting control (NTC). To each well was added 20 ng of pFmiR-ST6GAL1 or pFmiR-ST6GAL2 plasmids in 5 µl Opti-MEM (Gibco) and 0.1 µl Lipofectamine™ 2000 (Life Technologies) in 5 µl Opti-MEM (Gibco). The solution was allowed to incubate at room temperature for 20 min. HEK293T cells (25 µl per well, 400 cells/ µl in phenol red free DMEM with FBS 10%) were then added to the plate. Plates were incubated at 37°C, 5% CO_2. After 48 hours, the fluorescence signals of Cerulean (excitation: 433 nm; emission: 475 nm) and mCherry (excitation: 587 nm; emission: 610 nm) were measured using the clear bottom read option (SYNERGY H1, BioTek, Gen 5 software, version 3.08.01).

Data Analysis:

We calculated the ratio of Cerulean fluorescence over mCherry fluorescence (Cer/mCh) for each well in each plate. For each miRNA, triplicate values of the ratios were averaged and the standard deviation (S.D.) obtained. We calculated % error of measurement for each miRNA (100 × S.D./mean). As a quality control measurement (QC), we removed any plates or miRNAs that had high errors in the measurement (median error ±2 S.D. across all plates) and/or a high median error of measurement for the plate (>15% for ST6GAL1 and >14% for ST6GAL2). After QC we

obtained data for 2,161 miRNAs for ST6GAL1 and 2,166 miRNAs for ST6GAL2 out of 2601 total miRNAs screened. The Cer/mCh ratio for each miRNA was then normalized to the Cer/mCh ratio for the NTC *within that plate* and error was propagated. Data from all plates were then combined and z-scores calculated. A z-score of ±1.965, corresponding to a *two*-tailed *p*-value of 0.05, was used as a threshold for significance. Post-analysis we identified 69 miRNA hits for ST6GAL1 and 62 for ST6GAL2 (see Fig. 1, Supplementary Fig. 3 and Supplementary Data 1).

Western Blots: ST6GAL1 and ST6GAL2

Cells were seeded in six-well plates (80,000 cells/well) and cultured for 24 h in appropriate media. Cells were then washed with HBSS and transfected with miRNA mimics (50 nM mimic, Dharmacon, Horizon Discovery, 5 µL Lipofectamine 2000, Life Technologies in 250 µL OptiMEM). The media was changed to standard media 12 hours post-transfection. Cells were then lysed at 48 h post-transfection in cold RIPA buffer supplemented with protease inhibitors. For Western blot analysis, 50 µg of protein was run on 10% gels (SDS-PAGE) and transferred to iBlot2 Transfer Stacks (nitrocellulose, Invitrogen, catalog number: IB23002) using the iBlot2 transfer device (Invitrogen). Blots were incubated with Ponceau S Solution (Boston BioProdcuts, catalog #ST-180) for 10 min and the total protein levels were imaged using protein gel mode (Azure 600, Azure Biosystems Inc.). Blots were then blocked with 5% (PANC1, HT-29. OVCAR3) or 10% (A549) non-fat dry milk in TBST buffer (TBS buffer plus 0.1% Tween 20) for 1.5 hours at 55 rpm on rocker (LSE platform rocker, Corning) at room temperature. For ST6GAL1 blots were incubated with rabbit α-human-ST6GAL1 1° antibody (1:900 in TBST with 10% non-fat dry milk, catalog #: 14355-1-AP, Proteintech). For ST6GAL2, rabbit α-human-ST6GAL2 1° antibody (1:900 in TBST with 10% non-fat dry milk, catalog #: 28367-1-AP, Proteintech) was used. After overnight incubation at 4 ℃, blots were washed 4× for 2 min each with 0.1% TBST buffer. After washing, a secondary antibody was added (α- rabbit IgG-HRP, 1: 10,000 in TBST with 10% non-fat dry milk, Abcam). After incubation for 1 h at room temperature with shaking (60 rpm), blots were washed 4× for 2 min each with 0.1% TBST buffer. The blots were then developed using Clarity and Clarity Max Western ECL substrate according to the manufacturer's instructions (Bio-Rad). Membranes were imaged chemiluminescent mode (Azure 600, Azure Biosystems Inc.). Western blot analysis was conducted for ST6GAL1 up-miRs in four cell lines (A549, PANC1, HT-29, OVCAR3; miRs: miR-328-5p, -488-5p, -221-5p, -6883-5p, -5700, -765, -212-5p, -4430) and for ST6GAL1 down-miRs in three cell lines (A549, PANC1, HT-29; miRs: miR-6782-5p, -499a-5p, -216a-3p, -4531). For ST6GAL2, both up- and down-miRs were tested in two cell lines (A549, HT-29; up-miRs: miR-3619-5p, -124-3p, -605-3p, down-miR: miR-30c-2-3p, -6828-5p, -22-3p). All analysis was done in biological triplicate.

The α-human-ST6GAL1, 1° gave multiple bands in some cell lines. Therefore, we validated the antibody using the ON-TARGETplus siRNA reagent against ST6GAL1 in a smart pool format (Dharmacon, Horizon Discovery, CA) in PANC1 and A549 using the manufacturer's protocol (see Supplementary Fig. 4).

RT-qPCR:

Total RNA was isolated from cells treated as in Western blot experiments using TRIzol reagent (catalog #: 15596018, Invitrogen) according to the manufacturer's instructions. RNA concentrations were measured using NanoDrop, and high-quality isolated total RNA was reverse-transcribed to cDNA using Superscript III Cells Direct cDNA synthesis kit (catalog #: 18080300, Invitrogen). Reverse transcription quantitative PCR (RT-qPCR) was performed using the SYBR Green method and cycle threshold values (Ct) were obtained using an Applied Biosystem (ABI) 7500 Real-Time PCR machine and normalized to housekeeping gene GAPDH. The primer sequences used in RT-qPCR can be found in Table S1. All analysis was done in biological triplicate

SNA Staining Assay:

Cells were seeded onto sterile 22 × 22 no. 1 coverslips placed into 35 mm dishes at a density of 5 × 10^4 cells/ml in standard media. After 24h, cells were transfected with miRNA mimics or antimiRs as in the Western blot section. At 48 h post-transfection, cells were washed with PBS (3×, 2 mL) and fixed with 4 % paraformaldehyde for 15 min. Cells were again washed with PBS (3×, 2 mL). blocked using 10 % BSA in PBS for 1h in incubator (37 ℃, 5% CO_2) and Cy3-SNA was added (1:300 in 10 mM HEPES, 0.15 M NaCl, 0.1 mM $CaCl_2$, pH 7.5, Vector Laboratories, catalog # CL-1303). After 1 h in the incubator, coverslips were washed (PBS, 3×), and cells were counterstained with Hoechst 33342 (1 µg/mL in PBS, 15 min in incubator). The coverslips were then mounted onto slides with 60 µl of mounting media (90% glycerol in PBS) and imaged with a Zeiss fluorescent microscope (Camera: Axiocam 305 mono, software: ZEN 3.2 pro). For each biological replicate, 5 fields were obtained. Specificity of SNA staining was confirmed by using neuraminidase (gift from Dr. Matthew Macauley) prior to SNA staining. All analysis was done in biological triplicate. For data analysis, the ZOI method in the ZEN 3.2 pro software was used to quantify the fluorescence signal in membrane of all cells. Signal was normalized to cell count using the Hoechst staining to count nuclei in the software. Final data was normalized to the NTC for each biological replicate. A paired t-test was used to compare NTC with miRNA or anti-miR.

Endogenous miRNA Activity Validation:

miRIDIAN microRNA Hairpin Inhibitors (ST6GAL1: anti-miR-221-5p, anti-miR-212-5p, anti-miR-488-5p, anti-miR-765, anti-miR-499a-5p, anti-miR-4531; ST6GAL2: anti-miR-3619-5p, anti-miR-124-3p, anti-miR-6828-5p, anti-miR-30c-2-3p) and miRIDIAN microRNA Hairpin Inhibitor Negative Control (NTC) were purchased from Dharmacon (Horizon Discovery, Cambridge, UK). A549 cells were seeded and incubated as described for Western blot. A549 cells were transfected with anti-miRNAs, 50 nM using Lipofectamine™ 2000 transfection reagent in OptiMEM following the manufacturer's instructions (Life Technologies). After 12 h media was changed to standard culture media. 48 h post-transfection A549 cells were lysed and analyzed for ST6GAL1 and ST6GAL2 protein and mRNA levels as previously described. For ST6GAL1, anti-up-miRs (anti-miR-221-5p, anti-miR-212-5p, anti-miR-488-5p, anti-miR-765) were also tested in the PANC1 cell line. All analysis was done in biological triplicate.

Multi-Site Mutagenesis: ST6GAL1:

The 3'-UTR sequence of ST6GAL1 and the three miRNA sequences (miR-221-5p, miR-212-5p, miR-4531) were analyzed with the RNAhybrid tool which calculates a minimal free energy hybridization of target RNA sequence and miRNA. The two stable predicted miRNA: mRNA interaction sites were selected for designing mutant pFmiR-sensors. Multiple mutation sites were designed and mutant sequences were ordered for synthesis from GenScript Biotech or Integrated DNA Technologies (IDT). Each synthesized mutant fragment (221-MUTA-gBlock, 221-MUTB-gBlock, 212-MUTA-gBlock, 212-MUTB-gBlock) was amplified by standard PCR machine (Bio-Rad), using the primer sequences found in Table S1. Amplicons were cleaned up using Monarch PCR & DNA cleanup kit (catalog #: T1030S, NEB). The NucleoSpin Gel and PCR Clean-up XS kit (REF. #: 740611.50) was used for DNA gel extraction when needed to exclude non-specific bands. The amplicons were ligated into the empty pFmiR plasmid after enzymatic digestion using a pair of restriction enzymes for each gBlock (221-MUTA: NheI, PasI; 221-MUTB: PasI, BamHI; 212-MUTA: NheI, PasI; 212-MUTB: SwaI, BamHI). Sequences for the mutant pFmiR-ST6GAL1 sensors were then verified by sequencing and used in the miRFluR assay as described previously. A minimum of 3-wells were transfected per sensor and the analysis was done in 2 independent experiments.

Multi-Site Mutagenesis: ST6GAL2:

The 3'-UTR sequence of ST6GAL2 and the two miRNA sequences (miR-3619-5p, miR-30c-2-3p) were analyzed with the RNAhybrid tool which calculates a minimal free energy hybridization of target RNA sequence and miRNA. The two stable predicted miRNA: mRNA interaction sites were selected for designing mutant pFmiR-sensors. Multiple mutation sites were designed and mutant sequences were ordered for synthesis from GenScript Biotech or Integrated DNA Technologies (IDT). Each synthesized mutant fragment (3619-MUTA-gBlock, 3619-MUTB-gBlock, 30c-MUT-gBlock) was amplified by standard PCR machine (Bio-Rad), using the primer sequences found in Table S1. Amplicons were cleaned up using Monarch PCR & DNA cleanup kit (catalog #: T1030S, NEB). The NucleoSpin Gel and PCR Clean-up XS kit (REF. #: 740611.50) was used for DNA gel extraction when needed to exclude non-specific bands. The amplicons were ligated into the empty pFmiR plasmid after enzymatic digestion using a pair of restriction enzymes for each gBlock (3619-MUTA: AjuI, PasI; 3619-MUTB: PsiI-v2, PasI; 30c-MUT: AjuI, PasI). Sequences for the mutant pFmiR-ST6GAL2 sensors were then verified by sequencing and used in the miRFluR assay as described previously. A minimum of 3-wells were transfected per sensor and the analysis was done in 2 independent experiments.

siRNA Knock Down of microRNPs:

ON-TARGETplus siRNA reagents against AGO2, FXR1, TNRC6A in a smart pool format and ON-TARGETplus Non-Targeting Control Pool (NTP) were purchased from Dharmacon (Horizon Discovery, CA). A549 cells were seeded in six-well plates (50,000 cells/well) and cultured for 24 h in appropriate media. Cells were then washed with HBSS and transfected

with each of the siRNA pools (50 nM, NTP, AGO2, FXR1 or TNRC6A, Dharmacon, Horizon Discovery) with Lipofectamine™ RNAiMAX transfection reagent (catalog #: 13778150, Thermofisher) following the manufacturer's instructions. Media was changed 12 hours post-transfection. After 48 hours, cells were then transfected with miR-221-5p, miR-212-5p, or NTC

as previously described. Cells were then harvested for Western blot and RT-qPCR analysis as previously described. The knockdown efficiency for the siRNA was tested by Western blot analysis using 1:1000 dilution of 1° antibodies targeting AGO2 (catalog #: 67934-1-Ig, Proteintech), FXR1 (catalog #: 12295S, Cell Signaling) and TNRC6A (GW182) (catalog #: ab114857, Abcam) in 10 % non-fat dry milk in TBST (Figs. S11-12). Blots were processed as for ST6GAL1/2.

a

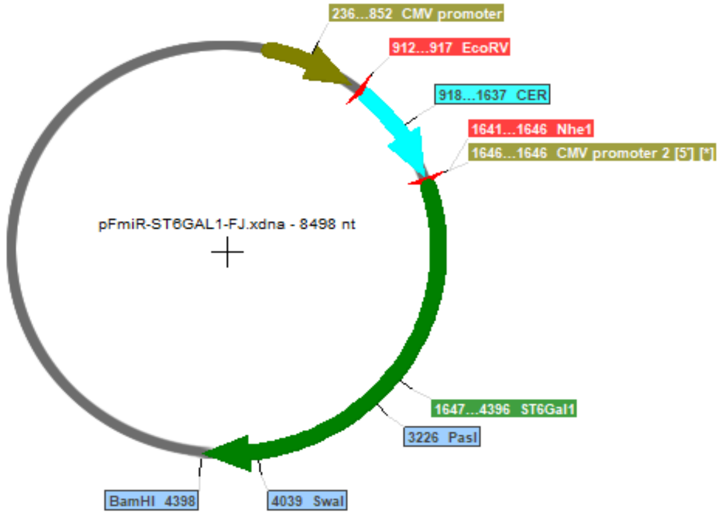

b

```
GCACAGGCTCCTCACTCTTCTCCATCAGGCATTAAATGAATGGTCTCTTGGCCACCCCAGCCTGGGAAGAACATTTTCCTGAACAATTCCAGCCT
GCTCCTTTTACTCTAGGGGCCTCTGTCAGCAAGACCATGGGGACTTCAAGAGCCTGTGGTCAGGAAATCAGGTCCAGCCTTCCCTGTAGCCAGAC
AGTTTATGAGCCCAGAGCCTCCTGCCACACACATGCACACATATCTAGCATTCTTTCCAGACAGCATCCTCCCCGCCTTCCACCTTGGTAGATGC
AAGGTCTATCTCTCCCATCAGGGCTGCCAAAGCTGGGCTTTGTTTTTCCCAGCAGAATGATGCCATTCTCACAAACCAATGCTCTATATTGCTTG
AAGTCTGCATCTAAATATTGATTTCACGTTTTAAAGAAATTCTCTTAAATTACAATTGTGCCCAATGCAGGGTGGCTCTGGGGGGCAAGTAGGTG
GTACAGGGGATTGGAAACATGCTCCGCGCCTCCAGAGAAAAGTTGCTCCCGAGGTCCATGCCCCTGGAACGTGTTCCTATCACTCTGGCTGGTTG
GGCTGGTCCTTAGACTGGGTGCTTATGATTAAAGGGTCTTGGTTAGCCCACTTTCCCTCTCCATGTGGAGATGGAAGGTAGAGAAGGATACAGTG
TCTATCCTCAAGTTGCTACGGTTCAGTGAGAGAGGCAGACATCTGAACAGGCAGGTAGGATTCAGTGTGCTCAGTGCACTGGGGATTTGGAGAGA
GATGGGCTTGCTCTCTCTGTGCACCCAGGAGGGCCACGCACTTAAAACTGTGTTTGTGGATCAGAGAAGGCTTTATAGCACAGGGGGCATTCAGA
TGAGTCTTAGAGGAAGAGAAGAAACATGGCAAGCAGATTACATCTGAGCCGTTTGAATTGTGTTTTTCTTTCTTCCCATGTTTATTTTCTAAGAT
CTACCTGAACTTAGAGACTCAAGATATTTTTTTAGGAAACCTCCTACCCATGTCTGAGGTAGCAAGTGCAGCCTCACGACAGATACCAGGCAATC
CAGAGCCACAAAACGTGATTCCTCCAGGCTCTGCCTGGCCTGACCCTGTCCTGTCAGCTGGGTTTACATACCAGTCCCATTCTTCCTTTTCAATA
CCTACCCCCAAATCTTCTCCTAACCACCATCTGTTTTTTTTTTTTAAAGCATTTTTTGCTTTAAAAGCATCCTGACCCCAATTTCTTTGAGCTCA
CGGGCCTTTTGCTGAAGGTCTCTCAGGGTGTAGTGGTGTGGCTCTCTGGACTTAACGTCACTCTCAGAGGTCAGAACCTTGGAGATCAGAACTGA
TTCTCACCAGGTGTGAGAGGTGTGGTAGCAGATTGCAATGCTCTGCACCTCTTCCTTGCAAGTGAGCAACTTCAGGCTCTCTGGGCAGAGGCTGG
CCCACTGTAGTTTGCAGACATGCTCTCCAGATGGTTTTACTAAGTCCCCTCTCCCTGATAGGGAATCCTGCTGGACCAGCGCAGCCCTGGTGTGG
AGAGGTTAAAAGACTTGCACAGGATCACCAAGTCATGCTGTAGAGCCAGGATTCCTAGACCCAGGGCTCTGCACTCTCAAGGCTGGCCCCATGTG
CTCAAGGGGATCTAATGTTTGGGCTCCAAACTAACCATCTCGGAGCTGGGCTCCTCATTTACTGCCAAACCCTCAGCTTATGTAGCTAGAAAGGG
CCCTGGAGTGAGAAAGCCTGGATTTTCAAATTGATGCTCCCCTACTGACTAGCTGTGCCACTCTGGGCAAATGCTCTTCCTTGAGCCTGTTTCCA
CACCTGTAAAGTGGGGATGATGATCCTATCTCACTGCTTTTGTGAGGATTACAGGAAAGCACCTGTCCTGGCTCTGTACCTGGCACGTAGTAGGT
GCTCAGTTCATGCTGGTTTCCTTCCTGCCTTTAGTAGGGACCTGCTCTGTGCTCACACCTCGGCTGCATGCACCCTGCTGTGACGGAGGCTAGTG
TGGAAGAGGTCCTGTCCTCAGGGAATTAACTGTCTTATTGGGAGACAACAACTGTCCTCCTTGGAACACCCAAGAAACCATGCAAAGCAGTGGAC
AACACAGAACACGCCCTCCTCCTCGCTGCCTGCAGCTCCAATCTGATTCTGCTTGGGAATGGGCGGAGCACGTGGGCTGCTTAACTGCTGTATAG
GACAAGCCCCTTACCCCTCTCTGGGCCCATGAATTCCTGGCTTGGTTTATGTTCTGATTTGACACACTGATTTTAATCTTCGAATCATGACACTG
AGTGCAGAGGAGGTGGCATTCCGACAGCAGGACATACATGTTGGTGTGAAGACTGGGACGACACTGGGTAGAATCTAGTTTTTAATTATTATTAA
TATAAAGGATCAAATTAATTTAAATATGATTCTGAAGTCTACAGAACTTTTAGTTCTGTGCTGTCTATGTGGACACTTTGGTAAAATGCAAATTA
TGATATGGACGTTATCATTGGTCTGGTGAGATGTTTCATATTTGTGACAGTTAATTTAAAAATTATGAGTTAATGCTGCCTGTGTCTATGGGGTT
CTGTCTTCTTTGATAGCCATCTATTCATCTGGATCATGGGACCCTCTCTAATCCTTCCACCAATCAAATAAGCTATTGCTATTGGTTTGGAGTTG
AGATATCAGTCTCGGAAACTTCTGAAAAATGCTAATAATTACCCAAGGATTATGTCAAATTTTAAAATAAATGTGTGTGTGTTTCTTTAA
```

Figure S1 | pFmiR-ST6GAL1. a, ST6GAL1-pFmiR plasmid map. **b**, ST6GAL1 3'-UTR sequence.

ST6GAL1

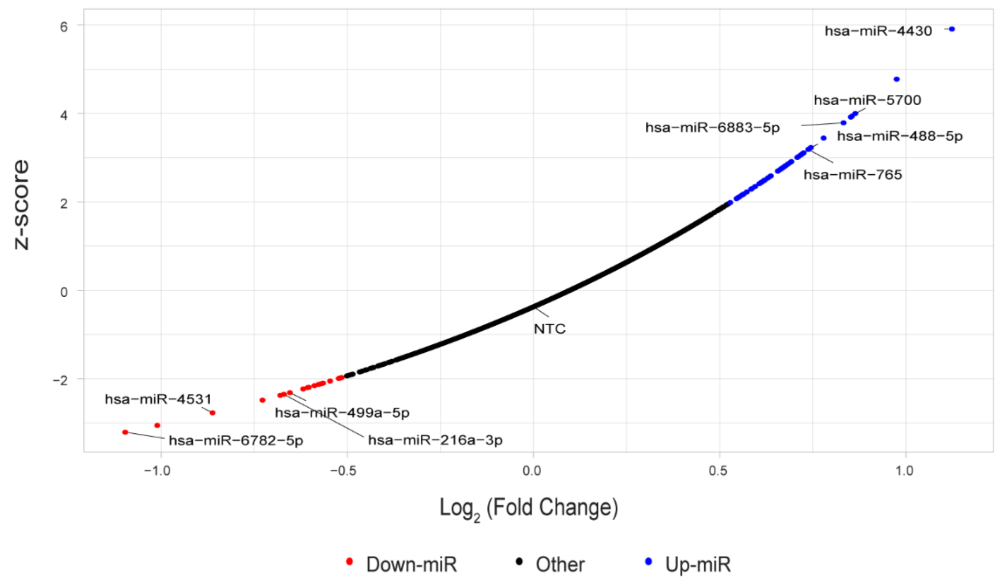

Figure S2 | Scatter plots of miRFluR assay showing up- and down-miRs. Data for ST6GAL1. miRNA in the 95% confidence interval are colored (down-miRs: red, up-miRs: blue).

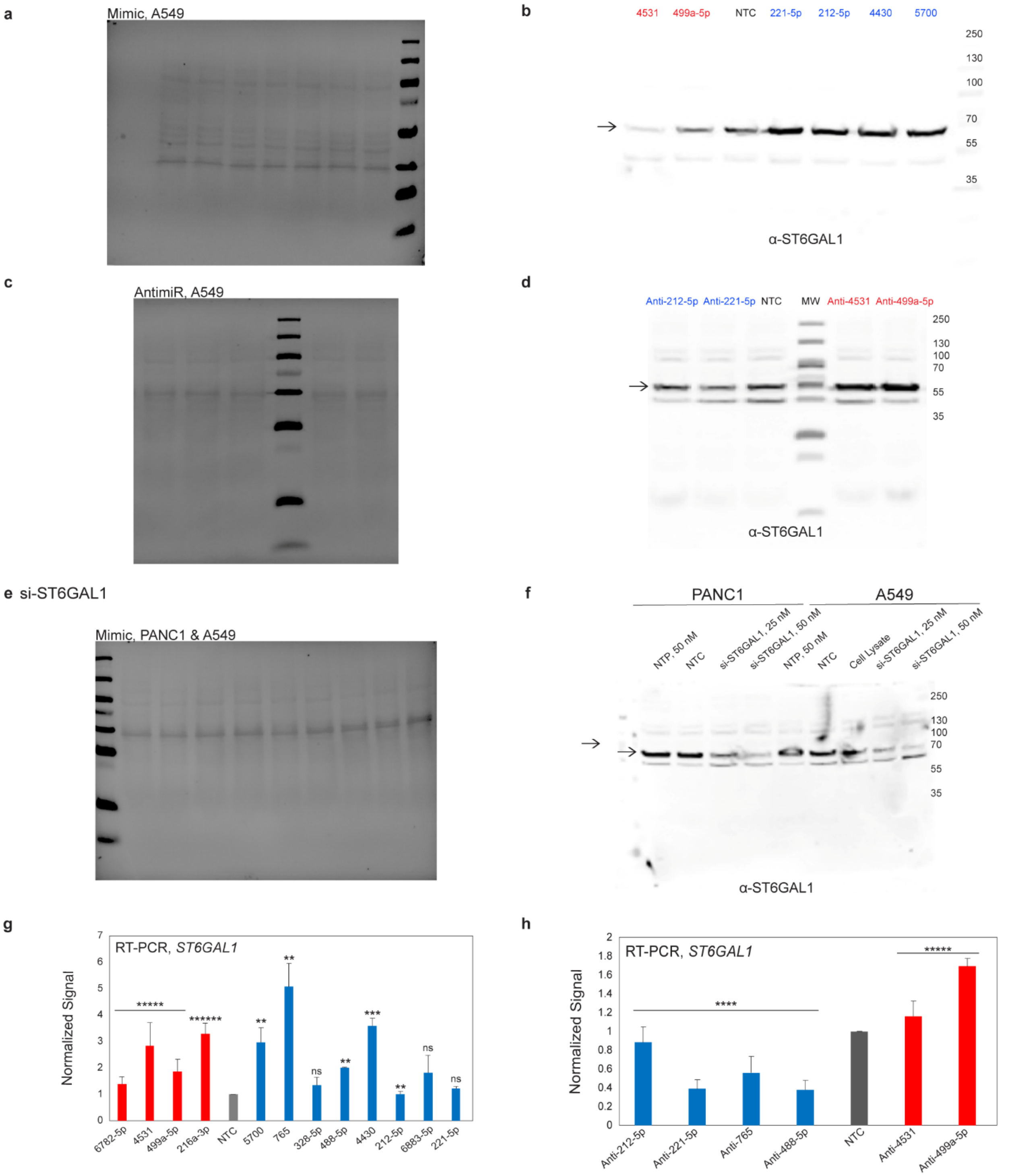

Figure S3 | miRNA up- and downregulate ST6GAL1 protein and mRNA expression in A549 human lung carcinoma cell line. a, Ponceau of Western blot shown in Figure 2b. **b**, Western blot shown in Figure 2b. **c**, Ponceau of Western blot shown in Figure 3b. **d**, Western blot shown in Figure 3b. **e**, Ponceau of Western blot shown in f. **f**, Validation of ST6GAL1 antibody. Pooled siRNA against ST6GAL1 were transfected into A549 and PANC1 using standard protocols. In all

ST6GAL1 Western blots arrow indicates the ~ 57 kDa ST6GAL1 protein validated by this knockdown. **g**, RT-qPCR analysis for samples as in Figure 2b-c. Data was normalized to GAPDH and to NTC. **h**, RT-qPCR for experiments as in Figure 3b-c. Samples were normalized to GAPDH and NTC. Paired t-test was used to compare miRs to NTC (^{ns} not significant, *$p < 0.05$, ** < 0.01, *** <0.001).

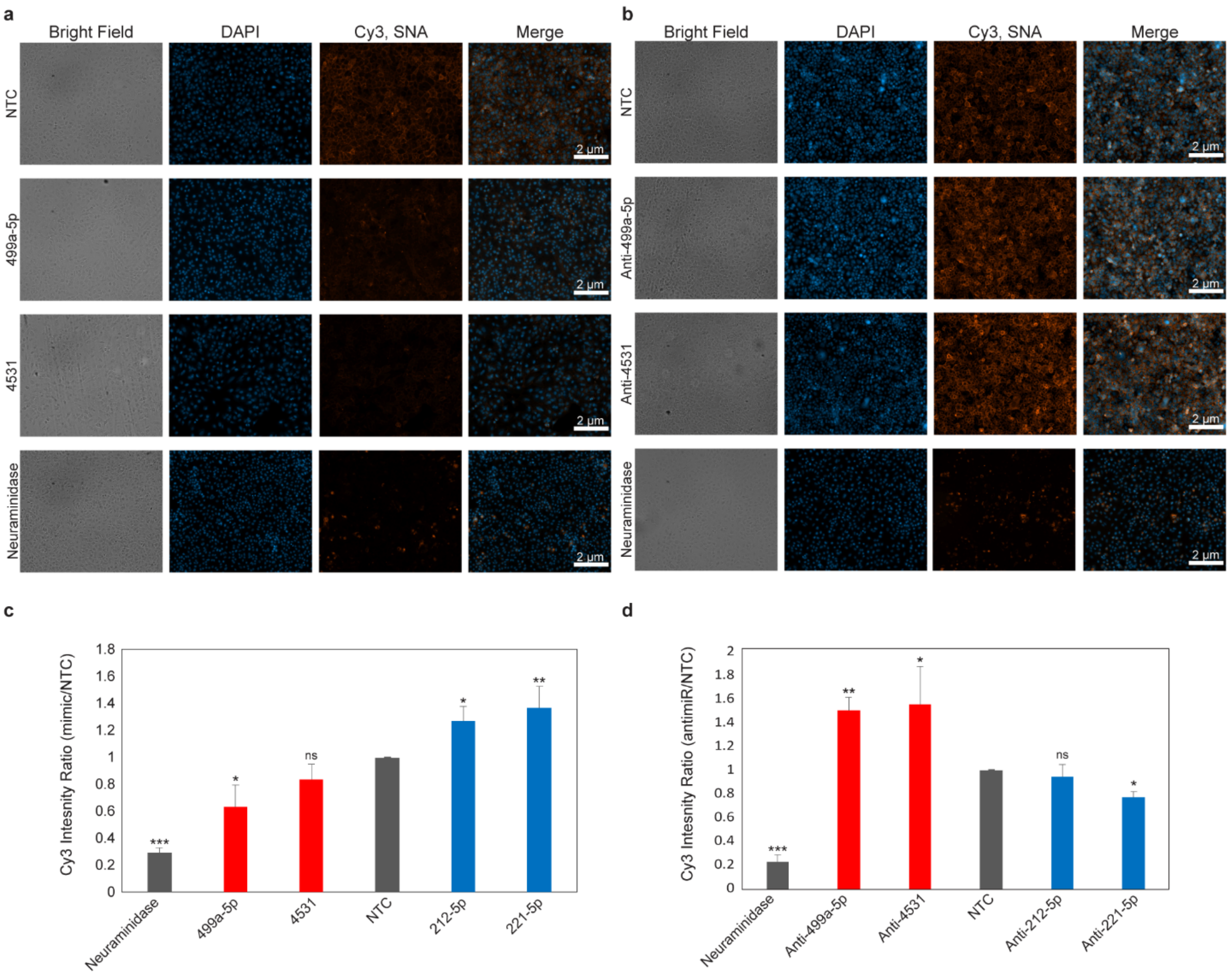

Figure S4 | miRNA impact α-2,6-sialylation in A549. a. SNA staining of A549 cells 48 h post-transfection with NTC or down miRs (miR-499a-5p, miR-4531). Neuraminidase treated cells are shown as a negative control. Our data shows ST6GAL1 protein downregulation by miRNAs correlates with decreased α-2,6-sialylation. **b**. SNA staining of A549 cells 48 h post- transfection with NTC or anti-down-miRs (anti-miR-499a-5p, anti-miR-4531). Images are representative of n=3 experiments. Our data shows downregulation of ST6GAL1 by anti-miRs correlates with increased α-2,6-sialylation activity. **c.** Quantification of Cy3 fluorescence signal representing α-2,6-sialic acid level on the membrane of cells transfected with NTC, down miRs (miR-499a-5p, miR-4531) or upmiRs (miR-221-5p, miR-212-5p), or neuraminidase treated cells. Cell count normalized data from 5 fields was gathered for each biological replicate. Data was then normalized to the NTC for that experiment. NTC ratios were then averaged for the 3 biological replicates and presented on the graph. **d.** Quantification of Cy3 fluorescence signal for corresponding antimiRs of miRs in c. Data analyzed as is c. Paired t-test was used to compare miRs to NTC (^{ns} not significant, * $p < 0.05$, ** < 0.01, *** < 0.001).

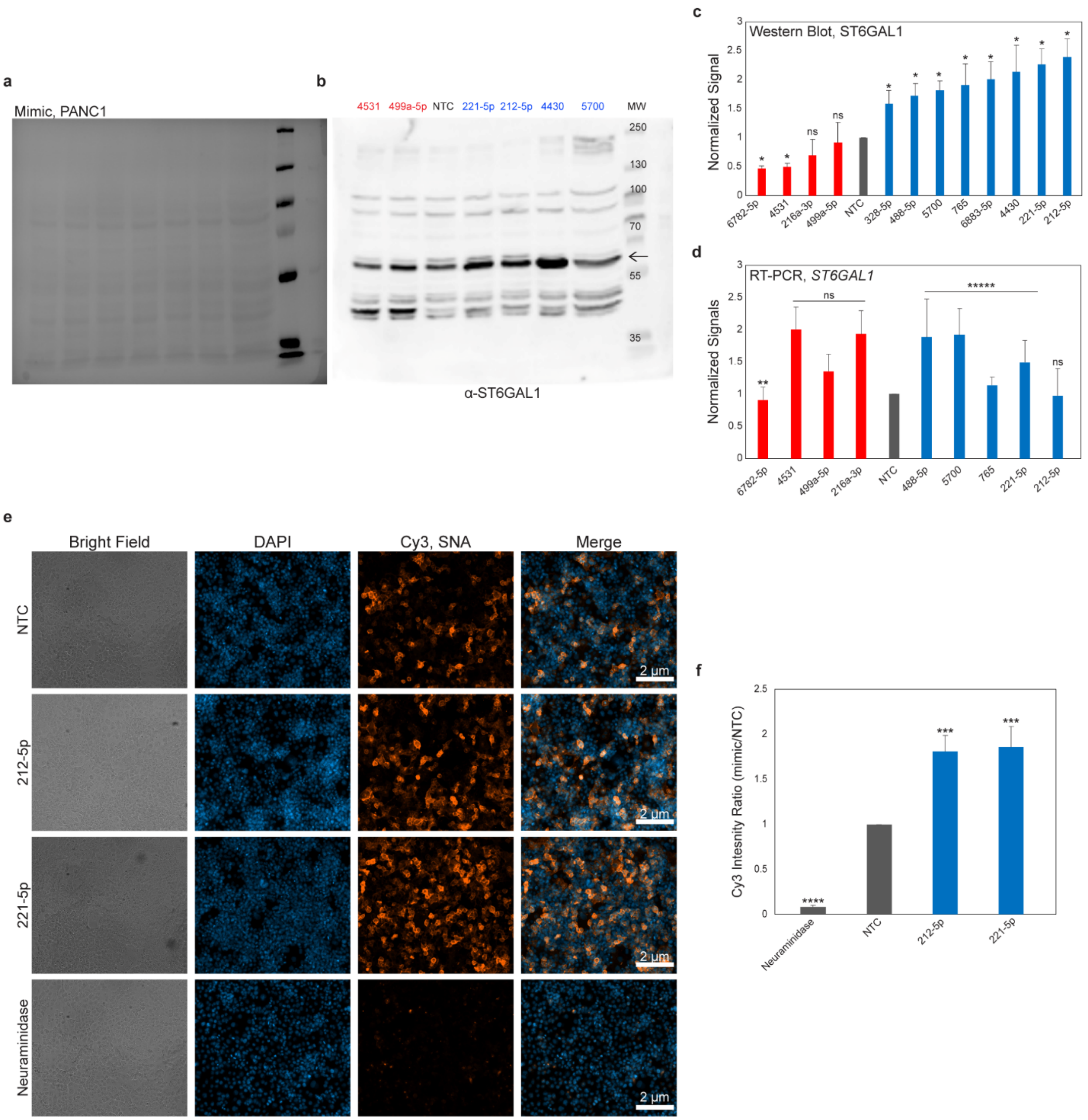

Figure S5 | miRNA up- and downregulate ST6GAL1 protein expression and α-2,6-sialylation activity in PANC1 pancreatic ductal carcinoma cell line. a. Ponceau of Western blot shown in b. **b**. Western blot analysis of ST6GAL1 in PANC1 transfected with representative 50 nM miR mimics or NTC, 48 h post-transfection (arrow indicates validated ST6GAL1 band). **c**. Quantitation of Western blot analysis illustrating up and downregulation of ST6GAL1 by individual miR mimics (up-miRs: miR-221-5p, 212-5p, -4430, -5700, -765, -328-5p, -6883-5p, -488-5p; down-miRs: miR-6782-5p, -499a-5p, -216a-3p, -4531; n=3). ST6GAL1 expression was normalized to total protein levels from Ponceau staining and set over normalized NTC for each blot.

d. RT-qPCR analysis for increased and decreased expression of ST6GAL1 mRNA by individual miR mimics. All samples are normalized to GAPDH as an endogenous housekeeping gene and then to NTC (n=3). **e**. SNA staining of PANC1 cells 48 h post-transfection with NTC, up-miRs (miR-212-5p, miR-221-5p). Neuraminidase treatment are shown as a negative control. Data

shown is representative of n=3 experiments. **f**. Quantification of Cy3 fluorescence signal representing α-2,6-sialic acid level on the membrane of cells transfected with NTC or upmiRs (miR-221-5p, miR-212-5p), or neuraminidase treated cells. Cell count normalized data from 5 fields was gathered for each biological replicate. Data was then normalized to the NTC for that experiment. NTC ratios were then averaged for the 3 biological replicates and presented on the graph. Paired t-test was used to compare miRs to NTC (^{ns} not significant, * $p < 0.05$, ** < 0.01, *** <0.001).

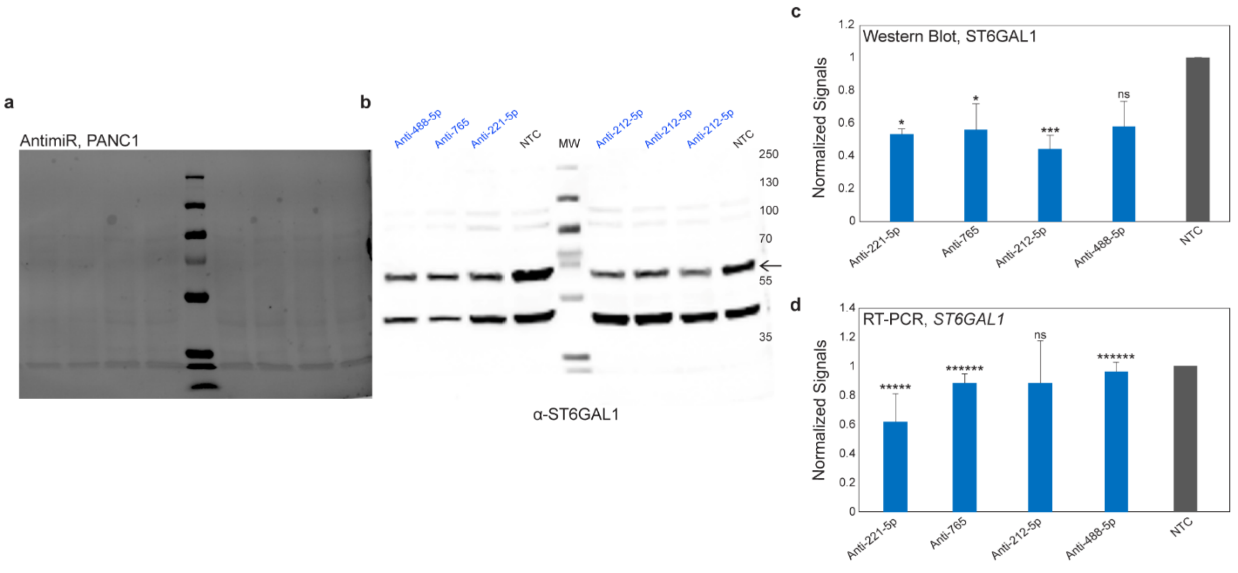

Figure S6 | Inhibiting endogenous up-miRs lowers ST6GAL1 protein expression levels in PANC1. a, Ponceau of Western blot shown in b. **b**, Western blot analysis of ST6GAL1 in PANC1 transfected with 50 nM anti-up-miRs (anti-221-5p, -212-5p, -488-5p, -765) or NTC, 48 h post-transfection (arrow indicates validated ST6GAL1 band). **c**, Quantitative Western blot analysis illustrating downregulation of ST6GAL1 protein by individual anti-up-miR (n=3). ST6GAL1 expression was normalized to total protein (Ponceau staining) and divided by normalized NTC for each blot. **d**, RT-qPCR analysis for decreased expression of ST6GAL1 mRNA by individual anti-up-miR. All samples are normalized to GAPDH as an endogenous housekeeping gene and then to NTC (n=3). Paired t-test was used to compare miRs to NTC (^{ns} not significant, * *p < 0.05, ** < 0.01, *** <0.001*).

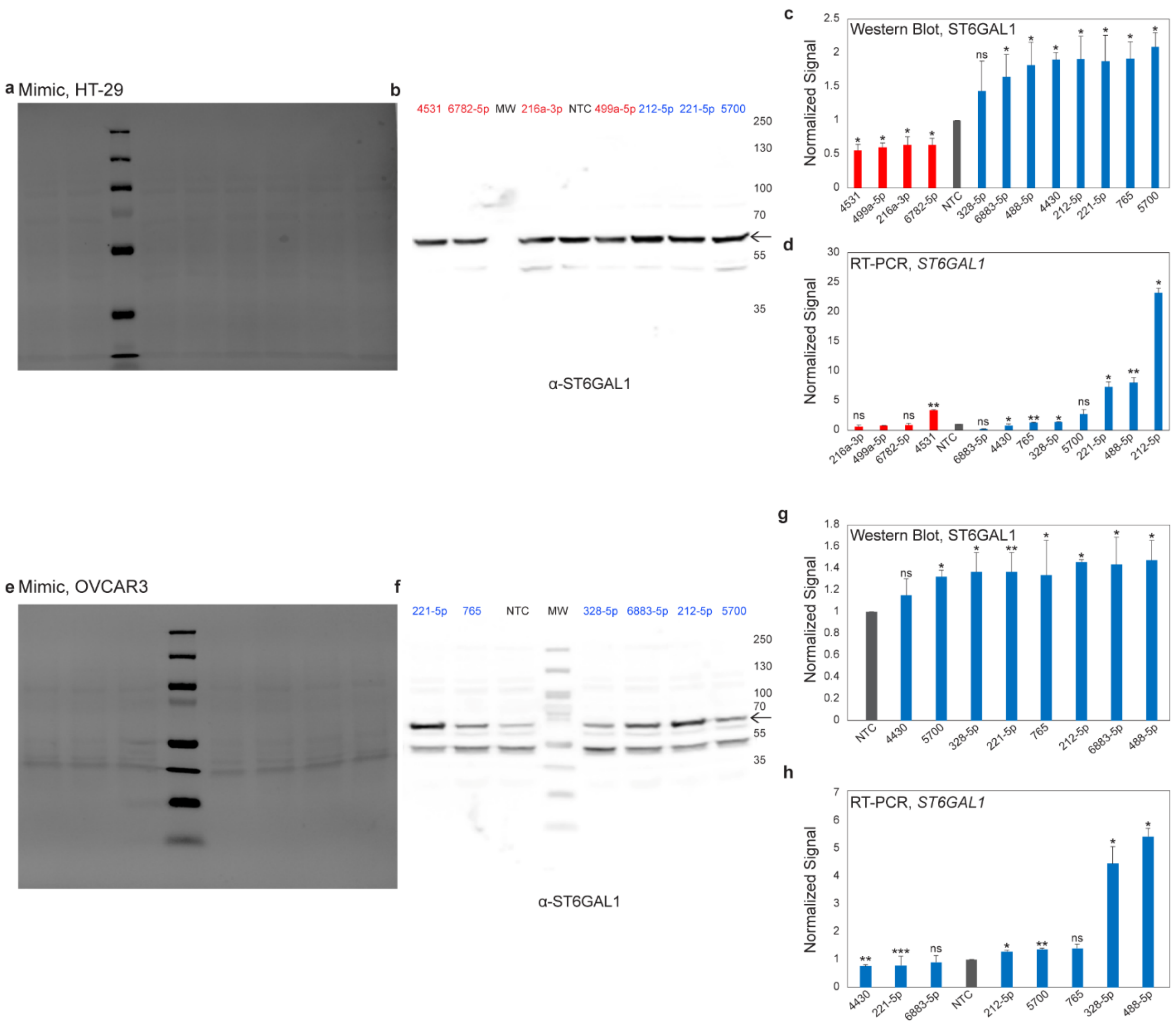

Figure S7 | miRNA up- and downregulate ST6GAL1 protein expression and α-2,6-sialylation activity in HT-29 human colorectal adenocarcinoma cell line and OVCAR3 high-grade serous ovarian adenocarcinoma cell line. a, Ponceau of Western blot shown in b. **b**, Western blot analysis of ST6GAL1 in HT-29 transfected with 50 nM miR mimics or NTC, 48 h post-transfection. A representative set is shown. **c**, Quantitative Western blot analysis illustrating up and downregulation of ST6GAL1 by individual miR mimics (n=3). ST6GAL1 expression was normalized to total protein (Ponceau staining) and divided by normalized NTC for each blot. **d**, RT-qPCR analysis of ST6GAL1 mRNA from cells treated as in c. All samples are normalized to GAPDH as an endogenous housekeeping gene and then to NTC (n=3). **e**, Ponceau of Western blot shown in f. **f**, Western blot analysis of ST6GAL1 in OVCAR3 transfected with 50 nM up-miR mimics or NTC, 48 h post-transfection. Representative miRNA are shown. **g**, Quantitative Western blot analysis illustrating upregulation of ST6GAL1 by individual up-miR mimics (n=3). ST6GAL1 expression was normalized as in c. **h**, RT-qPCR analysis of ST6GAL1 mRNA from cells treated as in g. All samples are normalized to GAPDH as an endogenous housekeeping gene and then to NTC (n=3). Arrows indicates validated ST6GAL1 band. Paired t-test was used to compare miRs to NTC (^{ns} not significant, * $p < 0.05$, ** < 0.01, *** < 0.001).

a

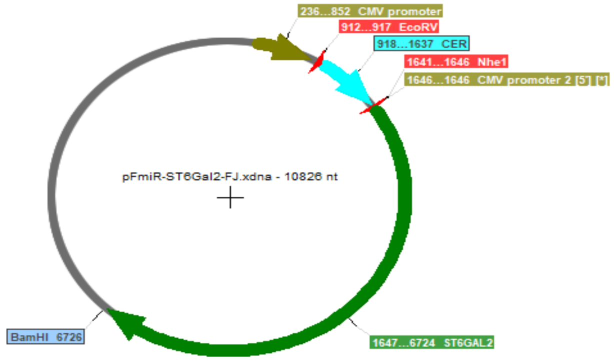


```
AAAGGGTTTCTTGGGAATCAATGTGCAATAAGGTACTACTGTTGTACTCAAAGTCACAAAAGAATACTTGAAGATAGATTTTAGACAGT
GTAGTTTTGAGTCTTTAAACAGTAATTTAGTGGTGGTCATGATAATCCTTCTCTTTCTAAGCCATTGAGTATGTATTACTGCTTTGAAC
ATAGAAATGGTTTTTTTAAAAAAATAAGGCTCAAGAAAGATGCAAAACCAAGGATAGCTAGAAAGAAAACACATGTATGACCATGGGTA
TGATACCTCCAGAAATGTTAACAACTTATTCTTCTGCCATGAGTACCCCTCATCAGGGTTGGTTTCAATGACAGGTTTTGGTGATGTTC
CTGACCATATGAAGTGGTTTATGTTTAGAAACATTCAAATTGAGGGACATCATTTACAGCATCGAGTGTGTCAGTTATACATGCATTCA
TCATTAAATATCCTAGGTTTTATGACAGACATTGAAATTATCACTCAGATGTGTTTAACAGCAAAAAATTCCCAGCACATCCTGGCAAA
GGCTTTTATTTCCAACCGTTGCATTCTTCATCTCTGCCTCCCATTGCCCACTGAATGCTTTGCTTTCTGTGCATCAAGACAGAGTTCTA
AAACCAGAAAACATCCATCTTGAAAGGTCCAAGAAAAAGCCAAATTCCATATAGCACCACAGAGGAGGCCCTTTGTTTCCCTGCAGTAT
TTCAACCAGGAACATTAGTATCACATCCACCAGGAGGCACATCACTGGCAAGTAACATAGAATCACAGGGCTGCAGGTGCCCCTGGACA
CTCCCCGGTTCTGGCTTTTCTGCCTTGTACCTCCTCACCTGCAGATCTACCCTGCAGCTTTTGCATAGTCAGTGACACACTTGACAATT
GGCACAGCTGTGCTGGGGAAGACGGGAGACACCTTGCTCCTTCCAAACTAGAAGGAAAGATTTTGGAGCCATCACCCTGTGCTTCTAGG
GATGCACGCCTGTCTTTATTATACAGGTTTCTTGCAGGTTTTTATCAGCCAGTGGGAACTCCACAGTGATGAGCACTGAATCTTCACTA
AATCTTCCTACAGACATTTAGGAATCCAGTCCTTGCTTTTGCAGATATGTTTTATGTCAGCAGCATGACAAAGCCATATAATTAAATAA
TTTATGTGAAAGCATTGCTTATTAACAAGGAAAATATGTAGATGCTTCAGAGGGAAAGGCAGCTAGAGGGAAATTTTCATCCCAATATG
TTAGCTATATTCAGGGTTGATTTTTTCTTTAATCTCAACTGTCACTGATACATTGAAAAATTTTTGCTATGAATATAGTGAAACAGAAA
CATTCTGTAATACCTTAATTAAACATGCTATTATTTCAATGTATAACCAAAACATATCTCCATGGTACAGTTAAGACTTGTTGCAATGA
CAATAGCAGCTTAATTAAATGCGTTCTCCCTTGCAAAATGTATGAATGTCAGGAGAGTTTAGTGGAAAAGTTTTCTTTGTTTCAGGATT
GGTTTCTTCTAGACTGTTCTCAGGACTACTTTCTTGAGCAAGAATGGCTTACAGAGACTGAAGGCTTCTGTTTGGTTAGTCTATTCAGC
AGATATGGAGCTGGAAAACAAGACATCTCCTATAGCTTGCTATAATAGTGAAGGAGAAATTGGAAAGTTTTAATCACAATAATATCCAA
TAAAAGCCCATTTTGAGAGTTTTGTTATTAAGTCAATGAGAAGGGATAGGTAACTTTGTCCTATATATTAACTTATGTCTAAATAGACA
TGGTTAAGCCATGTTCCTTTCTAATTTCTTTTCTTTTCTTTTTTTTTTTTTTTTTGAGACAGTCTCTCGCTCTGTCGCCCAGGCTAGA
GTGCAGTGGTGCGATCTTGGTTCACTGCCAGCTCTGCCTCCCACGTTCACACCATTCTCCTGCCTCAGCCTCCTGAGTAGCTGGGACT
ACAGGCACCCGCCACCACGCCCAGCTAATTTTTTGTATTTTTAGTAGAGACAGGGTTTCACCGTGTTAGCCAGGGTGGTCTCGATCTCC
TGACCTTGTGATCTGCCTGCCTCGGCCTCCCAAAGTGATGGGATTATAGGCGTGAGCCACCACCGCGCCCGGTCATTCCTTTCTAATTT
CTTGAAAAAACTTTGTCTTGTCCTTATGTTTACATGTGTGTAATAATTTCAGCAACTCTGGAATGTGGGTGAGGTGGCTGTGGTGGATG
AATATTACTCCCTACCCTCCTTGGATCTTTCTAGATCTTCCAGACCATTAAATGCCCCTGAAGCTCCTTTTATAGTTTTGCAAGTGGAT
TTACTTGTAGACATCCTTACTATCCAGCATAATCCCCCTTGGCTGACATGAGAACTTGGTTAGAGGAGAATTTATTGGGGTCAGTGTAA
ATATTCAAGTTCAAAAGCCTCAGAACCCAACACTTTAAAGATTTTTAAATGGAATTTTGTCGTAGTCATTGTACTTTTAAAATAGTACT
TTCAGTTTCATTGAGGTTACAAGAAAGACTGAAGAGTACAAAGGAAATTTAGTTACAATTTTTGCATTTATTATGGCCTTAAAGACTCT
TGGGAATCAGGCTCTGTTCAACAGTCAATAAATGTAATACACACATTAATATAGTACTGACTGGGCGTTCTTCTATAAAAGTTTAAGTT
CTTTGGCTTCTAAGAAAATGTTGGGCAGTTACAGTTAACTTGATGACTACTTCTAGTATTAGCCAAATGGGTCCTAAGTGGCAGCATTT
TGCCAGACATTGACTAAATCCTTACATGCCACATTTACTGAATTCCAATAAACATATAGTTCTTCTGTGGAGTAGTTAACAGTAAGTGG
CAGCTCACTGAATTTCTCCCAGGTTCTGTCTCTTCCAAAATCCTCCATCCTCTCTTATGAAGGGAAGCTGATAAAAAAAAATCCTCTTG
GTGCAAGCTGAAGCTGTTAAGGATTCCTTTTTTCACTCTCAGGACCAACAGACGTTCTTTTGTATTTCAAGATAATTTCACCTGTTGTT
GTTGTTGTTGTTGTTGTTGTTGTTGTTGTTGTTGTTGTTACAAAGAAACAAATTTTGAGCAGCAATGACAATTCAACCATATCTAGATT
CTCCCTACCTTATTTCTCCAGCCAGTCACACCAGGCAGACGGGTAACCTTTAACAGACCTGGTGTTCTCATGGAATACCGATGCTCGTC
CCAGGGAGCTGACCTTTCTCACTATGTCCTTATAGAGCTGCTCTTACAGAGAACTTTGTGCTCAGGACCAAGGGATGGTCAATTAAAAG
TTGCATGTAAGCATCAGGCTTGGATGGGCAAGGGAGGGCTTTTATACATCTTTGAACGACCAGGTCTTTCCTTGTCTGCCTGAAGGCAG
AGCTTCGAGAAACAGCACTTCCCATGTGTCTGGTCCTCTTGGTCTCTCAGCCAGACACTTAATCAATCCTGGACTCTCCAGTAGTCCTC
AGTCATTGTAGCCATAGCATCTGCAAAAAAAGAAATGCTAAAATAATCATTAGCGTTTTCAAAATTAGGAAATCAAAGTAGCTGGGTCA
CTCTCTTACATGTGTATGAGCCAGTTAATTCTGCTGGACAGATCATACTTACGCTGCTGATTGACTCTTCTGAGCAGTAACACATCTCC
TGAATTAATTAGGTAGGTCCCGTTTGGGTGCTTTAAGTGGGATCAGTCTTAGATGAGTGCCAGTATGTGATTGAATTTCATACGCACAA
ATGCCATGATTCAAGTTCTCTCTCCAGTTTTTCTTATAGATCAACACACAGTACATGTGGTCAGTTACTTGGTTGAATTTCTGCTTAGC
TGTGGTAAGAGACAGAGAAAGGGAGTGAGGTGGGACCAGGGGAATTATCAGAAAGACATGAGTGAACCTATTGGCCAGGTAGCTTCCAA
```

Figure S8 | pFmiR-ST6GAL2. a. ST6GAL2-pFmiR plasmid map. **b**. ST6GAL2 3'-UTR sequence.

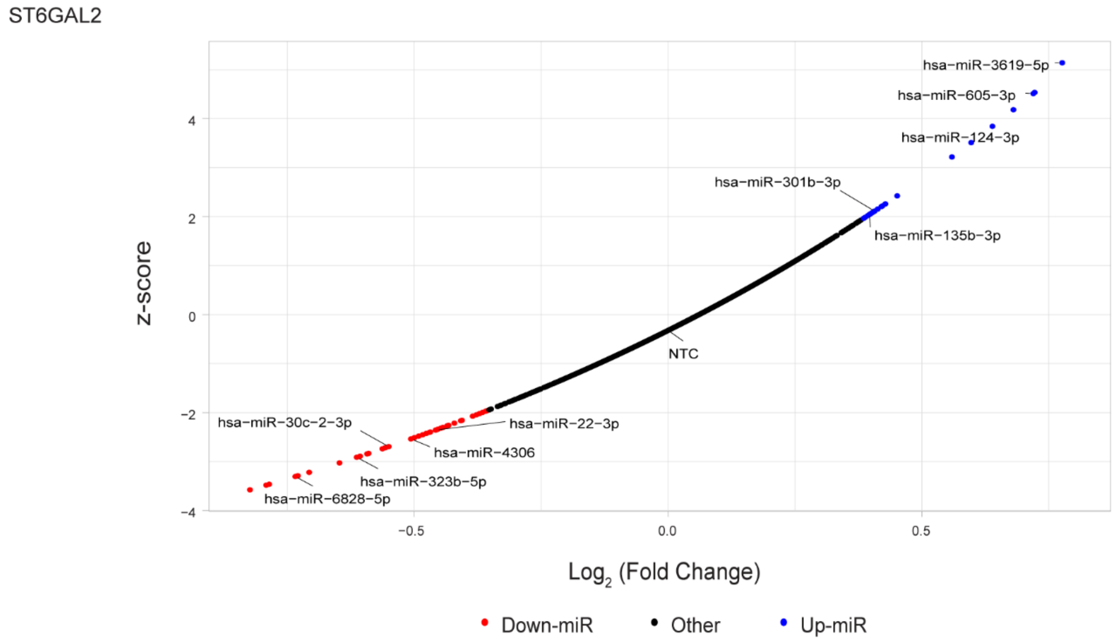

Figure S9 | Scatter plots of miRFluR assay showing up- and down-miRs. Data for ST6GAL2. miRNA in the 95% confidence interval are colored (down-miRs: red, up-miRs: blue).

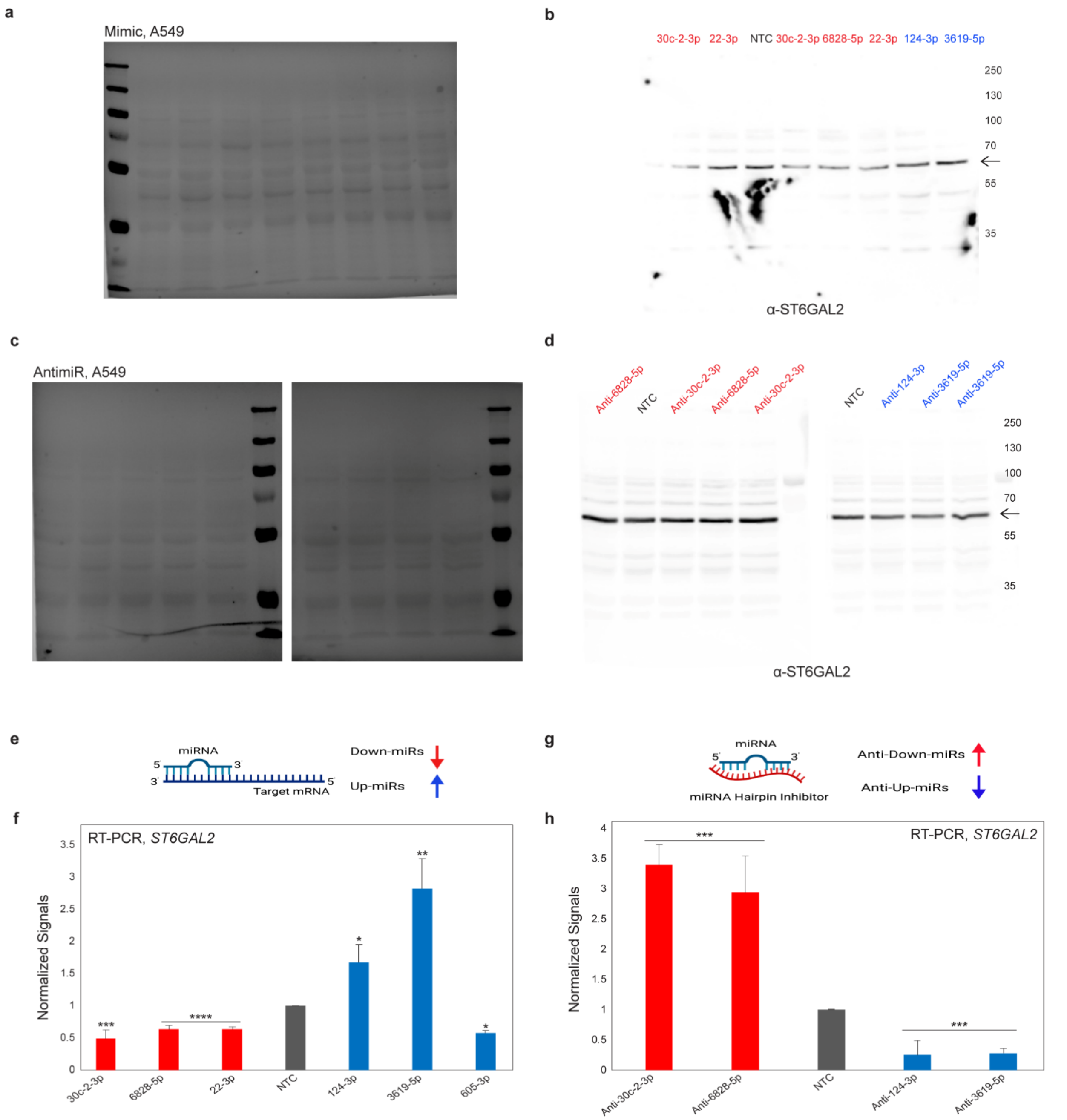

Figure S10 | miRNA up- and downregulate ST6GAL2 protein expression in A549 human lung carcinoma cell line. a, Ponceau of Western blot shown in Figure 4a. **b**, Western blot shown in Figure 4a. **c**, Ponceau of Western blot shown in Figure 4e. **d**, Western blot shown in Figure 4e. Arrows indicates ST6GAL2 band (~ 60 kDa). **e,** Scheme of miRNA regulation. **f,** RT-qPCR analysis corresponding to Figure 4d-e. **g,** Scheme of anti-miR regulation. **h**, RT-qPCR corresponding to Figure 4f-g. Paired t-test was used to compare miRs to NTC (^{ns} not significant, * $p < 0.05$, ** < 0.01, *** <0.001).

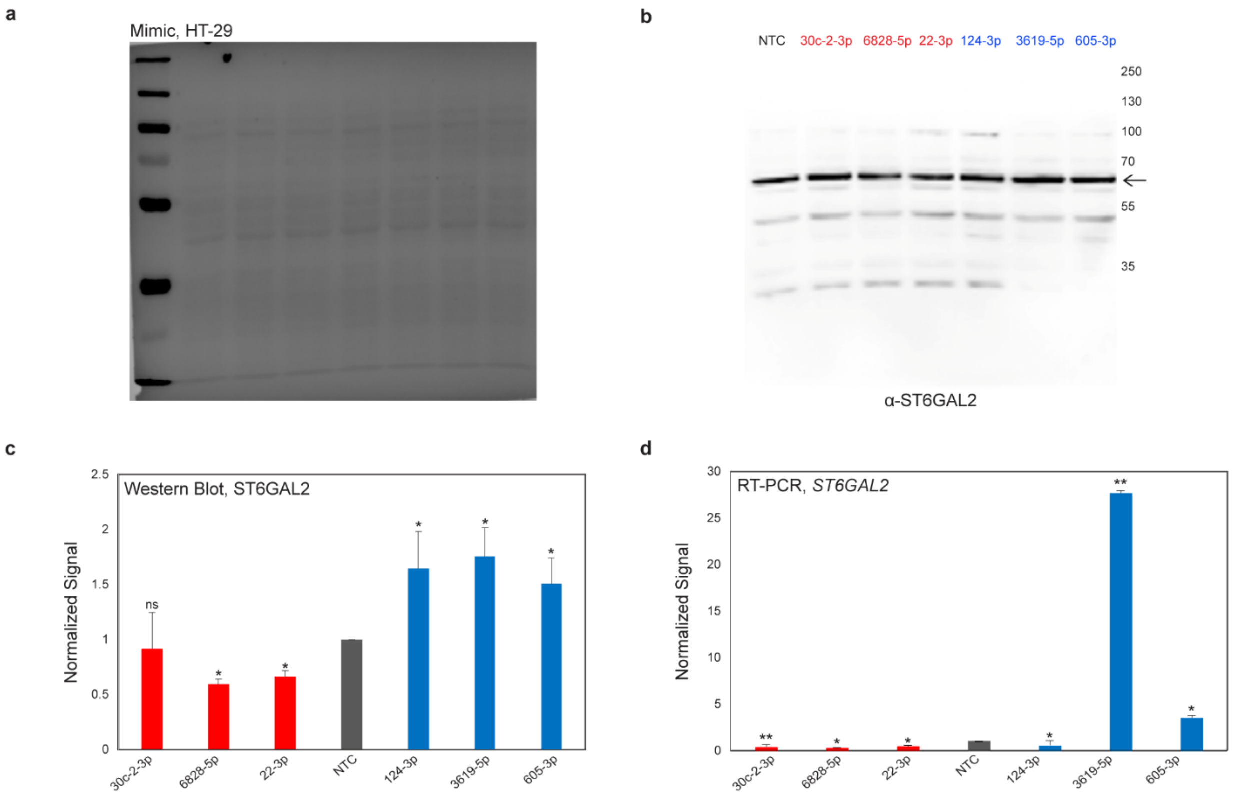

Figure S11 | miRNA up- and downregulate ST6GAL2 protein expression in HT-29 human colorectal adenocarcinoma cell line. a, Ponceau of Western blot shown in b. **b**, Western blot analysis of ST6GAL2 in HT-29 transfected with 50 nM miR mimics or NTC, 48 h post-transfection. Select subset of miRNAs is shown. Arrow indicates ST6GAL2. **c**, Quantitation of Western blot analysis illustrating up and downregulation of ST6GAL2 by individual miR mimics (up-miRs: miR-3619-5p, -124-3p, -605-3p, down-miRs: miR-30c-2-3p, -6828-5p, -22-3p). ST6GAL2 expression was normalized to total protein (Ponceau staining) and divided by normalized NTC for each blot. **d**, RT-qPCR analysis for samples as in c. All samples are normalized to GAPDH as an endogenous housekeeping gene and then to NTC. All experiments were done in triplicate. Paired t-test was used to compare miRs to NTC (^{ns} not significant, * p < *0.05, ** < 0.01, *** <0.001*).

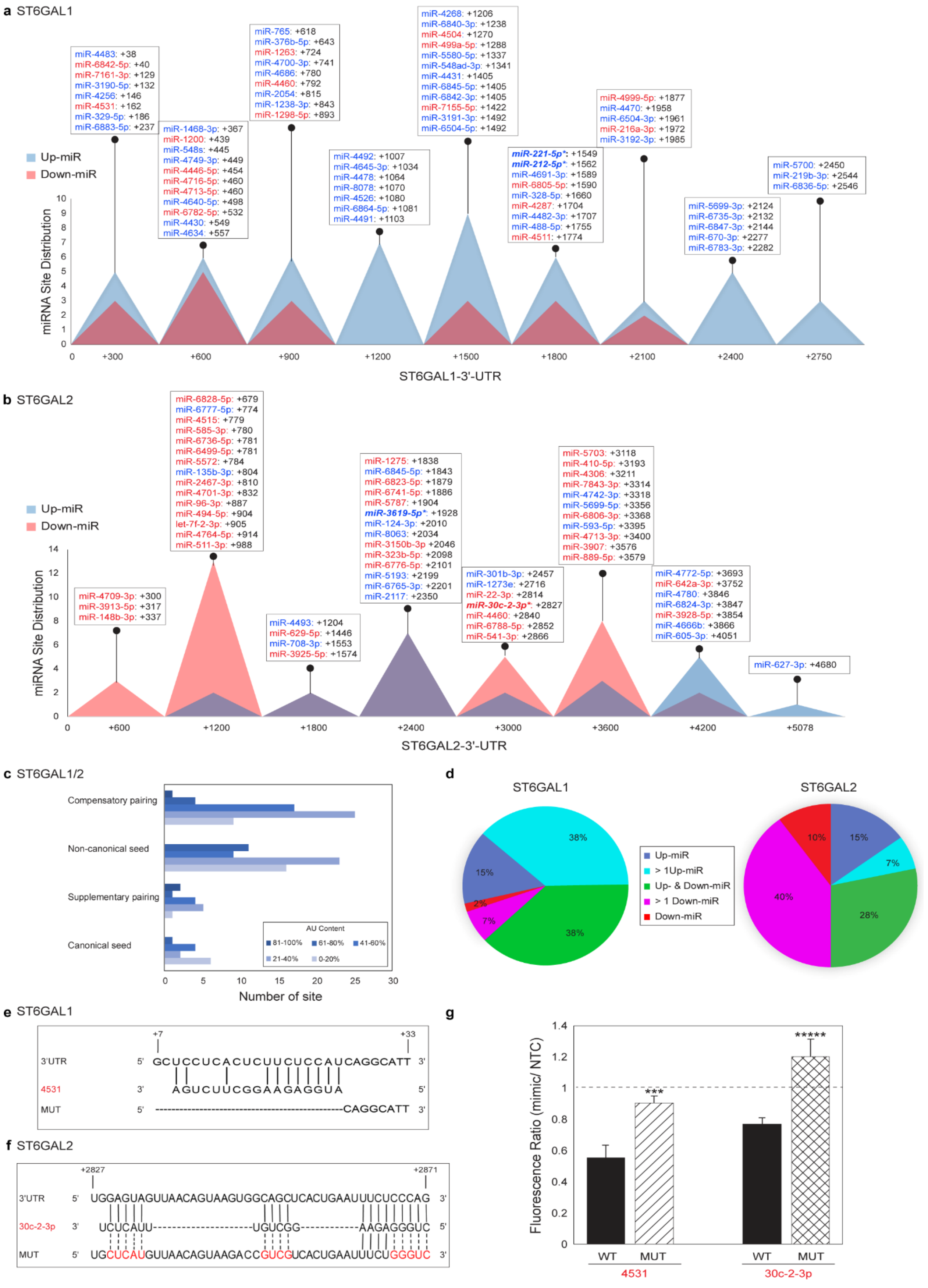

a ST6GAL1
miR-4483: +38
miR-6842-5p: +40
miR-7161-3p: +129
miR-3190-5p: +132
miR-4256: +146
miR-4531: +162
miR-329-5p: +186
miR-6883-5p: +237
miR-1468-3p: +367
miR-1200: +439
miR-548s: +445
miR-4749-3p: +449
miR-4446-5p: +454
miR-4716-5p: +460
miR-4713-5p: +460
miR-4640-5p: +498
miR-6782-5p: +532
miR-4430: +549
miR-4634: +557
miR-765: +618
miR-376b-5p: +643
miR-1263: +724
miR-4700-3p: +741
miR-4686: +780
miR-4460: +792
miR-2054: +815
miR-1238-3p: +843
miR-1298-5p: +893
miR-4492: +1007
miR-4645-3p: +1034
miR-4478: +1064
miR-8078: +1070
miR-4526: +1080
miR-6864-5p: +1081
miR-4491: +1103
miR-4268: +1206
miR-6840-3p: +1238
miR-4504: +1270
miR-499a-5p: +1288
miR-5580-5p: +1337
miR-548ad-3p: +1341
miR-4431: +1405
miR-6845-5p: +1405
miR-6842-3p: +1405
miR-7155-5p: +1422
miR-3191-3p: +1492
miR-6504-5p: +1492
miR-221-5p*: +1549
miR-212-5p*: +1562
miR-4691-3p: +1589
miR-6805-5p: +1590
miR-328-5p: +1660
miR-4287: +1704
miR-4482-3p: +1707
miR-488-5p: +1755
miR-4511: +1774
miR-4999-5p: +1877
miR-4470: +1958
miR-6504-3p: +1961
miR-216a-3p: +1972
miR-3192-3p: +1985
miR-5699-3p: +2124
miR-6735-3p: +2132
miR-6847-3p: +2144
miR-670-3p: +2277
miR-6783-3p: +2282
miR-5700: +2450
miR-219b-3p: +2544
miR-6836-5p: +2546
Up-miR
Down-miR
miRNA Site Distribution
0 1 2 3 4 5 6 7 8 9 10
0 +300 +600 +900 +1200 +1500 +1800 +2100 +2400 +2750
ST6GAL1-3'-UTR
b ST6GAL2
miR-4709-3p: +300
miR-3913-5p: +317
miR-148b-3p: +337
miR-6828-5p: +679
miR-6777-5p: +774
miR-4515: +779
miR-585-3p: +780
miR-6736-5p: +781
miR-6499-5p: +781
miR-5572: +784
miR-135b-3p: +804
miR-2467-3p: +810
miR-4701-3p: +832
miR-96-3p: +887
miR-494-5p: +904
let-7f-2-3p: +905
miR-4764-5p: +914
miR-511-3p: +988
miR-4493: +1204
miR-629-5p: +1446
miR-708-3p: +1553
miR-3925-5p: +1574
miR-1275: +1838
miR-6845-5p: +1843
miR-6823-5p: +1879
miR-6741-5p: +1886
miR-5787: +1904
miR-3619-5p*: +1928
miR-124-3p: +2010
miR-8063: +2034
miR-3150b-3p: +2046
miR-323b-5p: +2098
miR-6776-5p: +2101
miR-5193: +2199
miR-6765-3p: +2201
miR-2117: +2350
miR-301b-3p: +2457
miR-1273e: +2716
miR-22-3p: +2814
miR-30c-2-3p*: +2827
miR-4460: +2840
miR-6788-5p: +2852
miR-541-3p: +2866
miR-5703: +3118
miR-410-5p: +3193
miR-4306: +3211
miR-7843-3p: +3314
miR-4742-3p: +3318
miR-5699-5p: +3356
miR-6806-3p: +3368
miR-593-5p: +3395
miR-4713-3p: +3400
miR-3907: +3576
miR-889-5p: +3579
miR-4772-5p: +3693
miR-642a-3p: +3752
miR-4780: +3846
miR-6824-3p: +3847
miR-3928-5p: +3854
miR-4666b: +3866
miR-605-3p: +4051
miR-627-3p: +4680
Up-miR
Down-miR
miRNA Site Distribution
0 2 4 6 8 10 12 14
0 +600 +1200 +1800 +2400 +3000 +3600 +4200 +5078
ST6GAL2-3'-UTR
c ST6GAL1/2
Compensatory pairing
Non-canonical seed
Supplementary pairing
Canonical seed
AU Content
81-100%
61-80%
41-60%
21-40%
0-20%
0 5 10 15 20 25 30
Number of site
d
ST6GAL1
38%
15%
2%
7%
38%
Up-miR
> 1Up-miR
Up- & Down-miR
> 1 Down-miR
Down-miR
ST6GAL2
10%
15%
7%
40%
28%
e ST6GAL1
+7
+33
3'UTR 5' GCUCCUCACUCUUCUCCAUCAGGCATT 3'
4531 3' AGUCUUCGGAAGAGGUA 5'
MUT 5' ---CAGGCATT 3'
f ST6GAL2
+2827
+2871
3'UTR 5' UGGAGUAGUUAACAGUAAGUGGCAGCUCACUGAAUUUCUCCCAG 3'
30c-2-3p 3' UCUCAUU--------------------UGUCGG------------AAGAGGGUC 5'
MUT 5' UGCUCAUGUUAACAGUAAGACCGUCGUCACUGAAUUUCUGGGUC 3'
g
Fluorescence Ratio (mimic/ NTC)
0 0.2 0.4 0.6 0.8 1 1.2 1.4
***
*****
WT MUT
4531
WT MUT
30c-2-3p

Figure S12 | Predicted miRNA binding site analysis for α-2,6-sialyltransferases. a, Map of the binding sites for miRNA identified as hits by our miRFluR assay. Sites shown are the most stable hybridization sites predicted by RNAhybrid [19]. Annotations are given for every 300 bp. **b**, Map of the binding sites for miRNA identified as hits for ST6GAL2 by our miRFluR assay. Sites shown are the most stable hybridization sites predicted by RNAhybrid [19]. Annotations are given for every 600 bp. **c**, Bar graphs representing the percentage of AU content of four different predicted miR sites (canonical seed: perfect seed match, supplementary pairing: base pairing near to canonical site, non-canonical seed: imperfect seed match, compensatory pairing: base pairing near to non-canonical site) for all predicted up-miRs sites for ST6GAL1 and ST6GAL2. Sites considered in b are the most stable predicted sites based on RNAhybrid shown in a.
d, Pie charts representing the distribution of miRNA site overlap within the 3'-UTR for ST6GAL1 and ST6GAL2. Sites are defined as overlapping if the annotated hybridization sites share nucleotides. Periwinkle blue: up-miRs with no overlap, turquoise blue: overlap between 2 or more up-miRs, green: overlap between a set of up-miRs and down-miRs, magenta: overlap between 2 or more down-miRs, red: down-miRs with no overlap. Percent is function of sum of miRNA hits. **e** and **f**, Alignment of down-miRs (ST6GAL1: 4531, ST6GAL2: 30c-2-3p) with predicted 3'UTR sites and their corresponding mutants. **g,** Bar graph of data from mutant miRFluR sensors as in d and e. Data was normalized to NTC in each sensor. For statistics data was compared to wildtype (WT) for each miRNA. All experiments were performed in biological triplicate. Errors are standard deviations. Standard t-test was used (** $p < 0.05$, ** < 0.01, *** <0.001*).

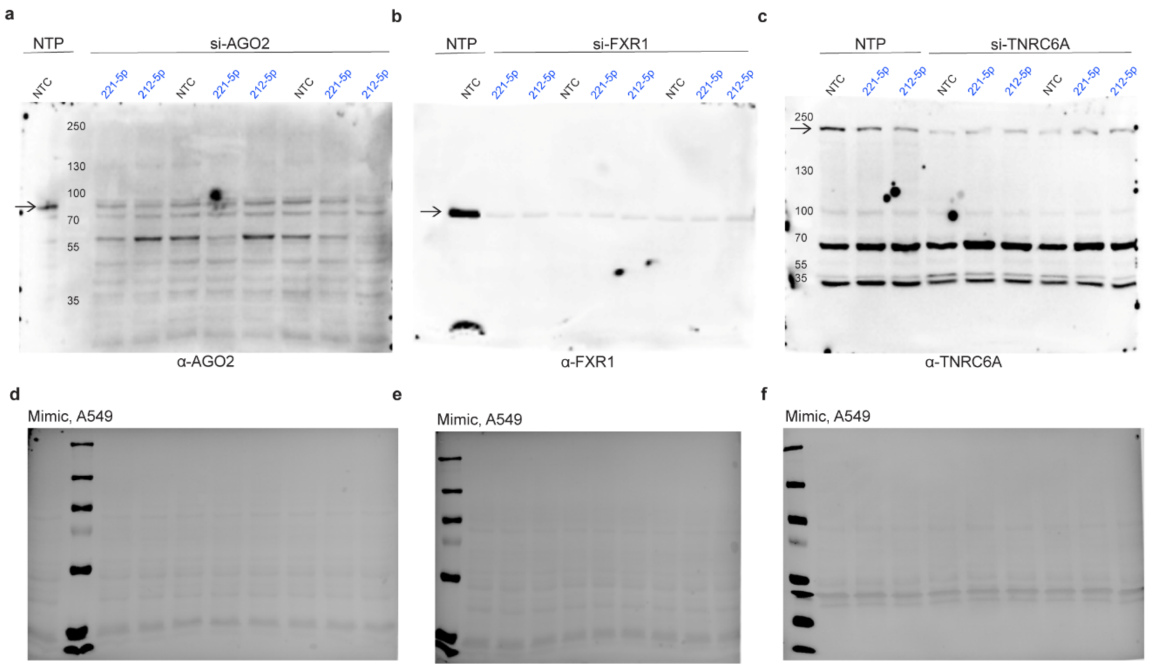

Figure S13 | Validation of siRNA knock down against AGO2, FXR1 and TNRC6A in A549 human lung carcinoma cell line for experiments shown in Figure 5.
a to **c**, Western blot analysis confirming knockdown of AGO2 (a), FXR1 (b) and TNRC6A (c) for samples used in Figure 5 and Supporting Information Figure S13. Arrows indicate the target bands. **d** to **f**, Ponceau of blots shown in a to c, respectively. Experiments shown are biological triplicates.

a

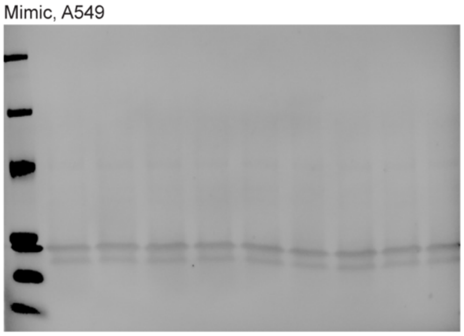
Mimic, A549

b

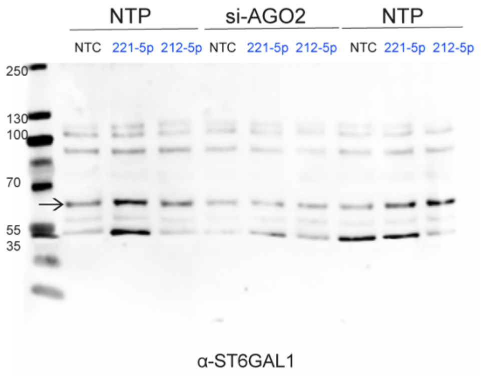
NTP
si-AGO2
NTP
NTC 221-5p 212-5p NTC 221-5p 212-5p NTC 221-5p 212-5p
250
130
100
70
55
35
α-ST6GAL1

c

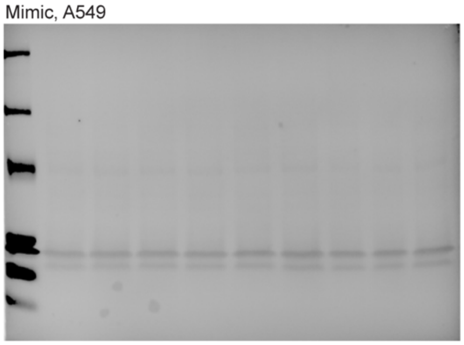
Mimic, A549

d

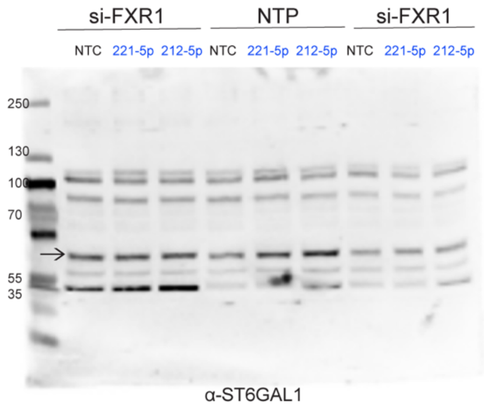
si-FXR1
NTP
si-FXR1
NTC 221-5p 212-5p NTC 221-5p 212-5p NTC 221-5p 212-5p
250
130
100
70
55
35
α-ST6GAL1

e

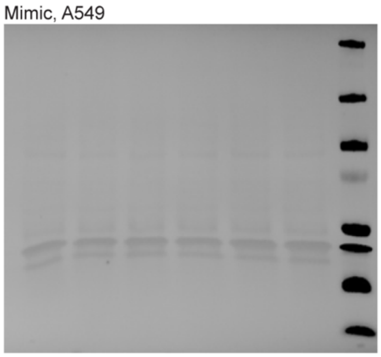
Mimic, A549

f

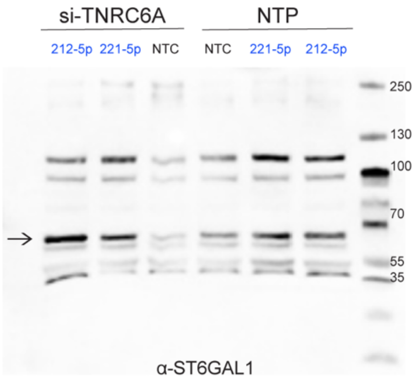
si-TNRC6A
NTP
212-5p 221-5p NTC NTC 221-5p 212-5p
250
130
100
70
55
35
α-ST6GAL1

g

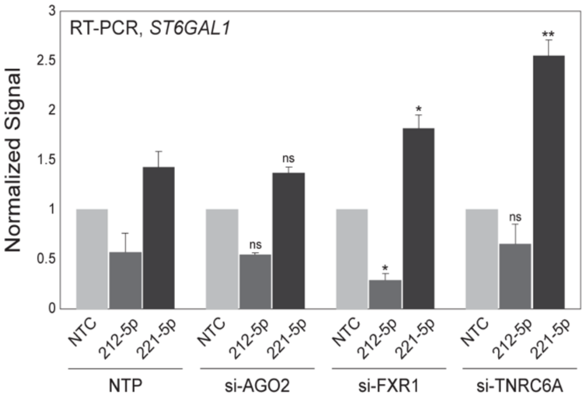
RT-PCR, ST6GAL1
Normalized Signal
3
2.5
2
1.5
1
0.5
0
ns
ns
*
*
ns
**
NTC 212-5p 221-5p NTC 212-5p 221-5p NTC 212-5p 221-5p NTC 212-5p 221-5p
NTP
si-AGO2
si-FXR1
si-TNRC6A

Figure S14 | Upregulation of expression by miRNAs requires FXR1 and AGO2 in A549 human lung carcinoma cell line. a, Ponceau of Western blots shown in b. **b**, Western blot of AGO2 shown in Figure 5b. **c**, Ponceau of Western blot shown in d. **d**, Western blot of FXR1 and NTP shown in Figure 5b. **e**, Ponceau of Western blot shown in f. **f**, Western blot of TNRC6A shown in Figure 5b. Arrows indicating the validated ST6GAL1 signal. **g**, RT-qPCR analysis for samples as in Figure 5g. All samples are normalized to GAPDH and NTC. All experiments were performed in biological triplicate. Errors are standard deviations. Standard t-test was used *(* $p <$ 0.05, ** < 0.01, *** <0.001*).

Primer Name	Sequence (5' → 3')	Sample
a. PCR amplification of ST6GAL1 and ST6GAL2 3'-UTRs		
ST6GAL1-FWD[a]	CGGACCATTCACTGCTAAG	gDNA, HEK293T
ST6GAL1-REV[a]	TTAAAGAAACACACACACATTTATTTA	gDNA, HEK293T
ST6GAL2-FWD	AAAGGGTTTCTTGGGAATC	gDNA, HEK293T
ST6GAL2-REV	TTCTAGACAAATGAAAACATG	gDNA, HEK293T
b. RT-qPCR[a] quantification of ST6GAL1, ST6GAL2 or GAPDH mRNA		
ST6GAL1-FWD1	GAACACCCAAGAAACCATGCA	Total RNA, A549 or HT-29
ST6GAL1-REV1	ACGTGCTCCGCCCATTC	Total RNA, A549 or HT-29
ST6GAL1-FWD2	AACACCCAAGAAACCATGCAA	Total RNA, PANC1 or OVCAR3
ST6GAL1-REV2	CGTGCTCCGCCCATTC	Total RNA, PANC1 or OVCAR3
ST6GAL2-FWD	GAAGGAGCCACGTGTTGGA	Total RNA, A549 or HT-29
ST6GAL2-REV	GCGGGTTCAGCATTTTGG	Total RNA, A549 or HT-29
GAPDH-FWD	GGTGTGAACCATGAGAAGTATGA	Total RNA, all cell lines
GAPDH-REV	GAGTCCTTCCACGATACCAAAG	Total RNA, all cell lines
c. PCR amplification of ST6GAL1 and ST6GAL2 mutant 3'-UTRs		
221-MUTA-FWD	CGGACCATTCACTGCTAAG	221-MUTB-gBlock
221-MUTA-REV	TCTAGGAATGGACCGTCTACT	221-MUTB-gBlock
221-MUTB-FWD	CTCTGCACTCTCAAGGC	221-MUTA-gBlock
221-MUTB-REV	TTAAAGAAACACACACACATTTAT	221-MUTA-gBlock
212-MUTA-FWD	CGGACCATTCACTGCTAAG	212-MUTB-gBlock
212-MUTA-REV	AGCTCCGAGATGGTTAGTTTG	212-MUTB-gBlock
212-MUTB-FWD	ATGATTCTGAAGTCTACAGAAC	212-MUTA-gBlock
212-MUTB-REV	TTAAAGAAACACACACACATTTAT	212-MUTA-gBlock
4531-MUT-FWD	CAGGCATTAAATGAATGGTCTCT	pFmiR-ST6GAL1
4531-MUT-REV	TTAAAGAAACACACACACATTTAT	pFmiR-ST6GAL1
3619-MUTA-FWD	CACCCTGTGCTTCTAGGGATGCACGCCT	3619-MUTA-gBlock
3619-MUTA-REV	ACGAGCATCGGTATTCCATGA	3619-MUTA-gBlock
3619-MUTB-FWD	AGCTGACCTTTCTCACTATG	3619-MUTB-gBlock
3619-MUTB-REV	ATGTTAGATTTTAACACCACCAATGC	3619-MUTB-gBlock
30c-MUT-FWD	CTTCTAGGGATGCACGCCTG	30c-MUT-gBlock
30c-MUT-REV	ACGAGCATCGGTATTCCATGAG	30c-MUT-gBlock

[a] FWD, forward; REV, reverse; RT-qPCR, Reverse transcription quantitative polymerase chain reaction.

Table S1. Primer sequences for PCR amplification of WT 3'-UTRs (a), RT-qPCR quantification of mRNAs (b), mutant 3'-UTRs (c) for ST6GAL1 and ST6GAL2.

Data S1. (separate excel file) | miRFluR results for ST6GAL1. Data analysis before and after thresholds.

Data S2. (separate excel file) | miRFluR results for ST6GAL2. Data analysis before and after thresholds.
